# Miniscope-LFOV: A large-field-of-view, single-cell-resolution, miniature microscope for wired and wire-free imaging of neural dynamics in freely behaving animals

**DOI:** 10.1126/sciadv.adg3918

**Published:** 2023-04-21

**Authors:** Changliang Guo, Garrett J. Blair, Megha Sehgal, Federico N. Sangiuliano Jimka, Arash Bellafard, Alcino J. Silva, Peyman Golshani, Michele A. Basso, Hugh Tad Blair, Daniel Aharoni

**Affiliations:** ^1^ David Geffen School of Medicine, University of California, Los Angeles, Los Angeles, CA 90095, USA.; ^2^ Department of Neurology, David Geffen School of Medicine, University of California, Los Angeles, Los Angeles, CA 90095, USA.; ^3^ Integrative Center for Learning and Memory, University of California, Los Angeles, Los Angeles, CA 90095, USA.; ^4^ Brain Research Institute, University of California, Los Angeles, Los Angeles, CA 90095, USA.; ^5^ Department of Psychology, University of California, Los Angeles, Los Angeles, CA 90095-1563, USA.; ^6^ Center for Neural Science, New York University, New York, NY 10003, USA.; ^7^ Department of Psychiatry and Biobehavioral Sciences, University of California, Los Angeles, Los Angeles, CA 90095, USA.; ^8^ Department of Neurobiology, University of California, Los Angeles, Los Angeles, CA 90095, USA.; ^9^ Jane and Terry Semel Institute for Neuroscience and Human Behavior, University of California, Los Angeles, Los Angeles, CA 90095, USA.; ^10^ West LA Veterans Affairs Medical Center, Los Angeles, CA 90073, USA.; ^11^ Intellectual and Developmental Disabilities Research Center, University of California, Los Angeles, Los Angeles, CA 90095, USA.

## Abstract

Imaging large-population, single-cell fluorescent dynamics in freely behaving animals larger than mice remains a key endeavor of neuroscience. We present a large-field-of-view open-source miniature microscope (MiniLFOV) designed for large-scale (3.6 mm × 2.7 mm), cellular resolution neural imaging in freely behaving rats. It has an electrically adjustable working distance of up to 3.5 mm ± 100 μm, incorporates an absolute head orientation sensor, and weighs only 13.9 g. The MiniLFOV is capable of both deep brain and cortical imaging and has been validated in freely behaving rats by simultaneously imaging >1000 GCaMP7s-expressing neurons in the hippocampal CA1 layer and in head-fixed mice by simultaneously imaging ~2000 neurons in the dorsal cortex through a cranial window. The MiniLFOV also supports optional wire-free operation using a novel, wire-free data acquisition expansion board. We expect that this new open-source implementation of the UCLA Miniscope platform will enable researchers to address novel hypotheses concerning brain function in freely behaving animals.

## Teaser

A large field of view open-source miniature microscope is capable of imaging large populations of neurons in rats and mice.

## INTRODUCTION

Understanding how populations of neurons and underlying circuits give rise to complex behavior remains a key endeavor of systems neuroscience. Novel imaging tools combined with the development of new calcium (Ca^2+^) indicators have advanced neuroscience by introducing the ability to monitor large populations of neurons simultaneously and track identified neurons across weeks to months (*1*). Decades of research have led to the development and refinement of optical techniques, such as multiphoton (*2*–*4*), confocal (*5*), and light sheet microscopy (*6*) to image the structures and functions of large neuronal networks with cellular and subcellular resolutions (*7*). However, many of these approaches require bulky imaging devices that can only be used on head-restrained animals (*8*, *9*), constraining experiments from being conducted in more naturalistic environments and behaviors.

Single-photon epifluorescence miniature microscopy (*10*, *11*) and multiphoton miniature microscopy (*12*–*16*) circumvent the constraint of head stabilization while still achieving single-cell resolution, enabling these optical techniques in freely behaving animals and expanding the repertoire of behavioral assays that can be used in conjunction with neural imaging. Open-source head-mounted miniaturized epifluorescence microscopes, such as the open-source UCLA Miniscope (*17*–*19*), FinchScope (*20*), miniScope (*21*), NiNscope (*22*), CHEndoscope (*23*), MiniLFM (*24*), Miniscope3D (*25*), and counterpart miniscopes (*26*, *27*), have shown that miniaturized microscopes are light enough to be mounted on the head of a rodent and record behaviorally relevant neural signals extracted by further analytical techniques (*28*–*30*). These developments over the past decade have been used extensively in freely behaving animals to reveal neural dynamics related to learning and memory (*17*), neurological disorders (*19*), and social interactions (*31*).

Previous designs of UCLA Miniscopes were developed specifically for mice, which resulted in a field of view (FOV) of ~1 mm^2^, limiting their capabilities and applications in imaging large-scale, single-cell fluorescent dynamics in larger animals (*32*–*34*). Furthermore, Ca^2+^ indicators generally have lower expression and fluorescence in animals other than mice, motivating the need for a high-sensitivity, large-FOV imaging system with single-cell resolution. In addition, current wired miniature microscopy devices lack optional configurations for wire-free operation, impeding experiments that involve recording multiple interacting animals simultaneously or animals navigating large environments (*31*, *35*–*37*).

Here, we report a head-mounted, open-source large-FOV miniscope (MiniLFOV) developed as a new implementation of the UCLA Miniscope platform. It has two optical configurations optimized at different working distances (WDs) for superficial and deep neural imaging. A 1.8-mm-WD configuration provides 2.5-μm (center) to 4.4-μm (edge) resolution across 3.1 mm × 2.3 mm FOV, and a 3.5-mm-WD enables 3.5-μm (center) to 6.2-μm (edge) resolution across 3.6 mm × 2.7 mm FOV. With a high-sensitivity 5-MP monochrome complementary metal-oxide semiconductor (CMOS) image sensor, the MiniLFOV yields 20-fold better sensitivity than the previous generation Miniscope V3, and twice the sensitivity compared to the current generation Miniscope V4. The WD is electrically adjustable to cover 1.8 mm/3.5 mm ± 100 μm depth focus using an electrowetting lens (EWL). Incorporated with a nine-axis absolute orientation sensor, the MiniLFOV has the ability to collect head movement data at up to 100 Hz, which can help investigate head orientation–related neural mechanisms (*38*).

The MiniLFOV is 35-mm tall, weighs 13.9 g, and can be modified for different experiments by swapping its custom objective module. The two WD configurations enable multiple applications such as cortical imaging through a cranial window and deep brain imaging using one or multiple implanted optical probes. Furthermore, we also developed a small, wearable wire-free data acquisition (DAQ) expansion board (wire-free DAQ, 3.5 g) which adds wire-free capabilities to all generations of coaxial cabled UCLA Miniscopes. The wire-free DAQ is battery-powered by an on-board, single-cell lithium-polymer battery and records imaging data onto an on-board microSD card. The wire-free DAQ can be mounted directly onto the MiniLFOV’s housing or be worn as a backpack. Power, bidirectional configuration commands, and high-bandwidth unidirectional imaging data are transmitted between the MiniLFOV and wire-free DAQ via a short coaxial cable. Integrated with an infrared (IR) remote control receiver, the system supports remote triggering with an IR remote control transmitter.

We validated the wired MiniLFOV in freely behaving rats running on a large rectangular track by imaging GCaMP7s-expressing neurons in the hippocampal CA1 pyramidal layer using a 1.8-mm-diameter relay gradient refractive index (GRIN) lens (FOV = ~2.54 mm^2^) (*24*, *39*, *40*), with more than 1300 cells imaged in a single 15-min session. We also validated the wire-free configuration of the MiniLFOV in rats exploring an open circular field. In addition, we validated the MiniLFOV in head-fixed mice on a circular treadmill by imaging GCaMP6f-expressing neurons in dorsal cortex through a cranial window with more than 1900 neurons recorded in a single 7-min session. With greater than a 30-fold increase in FOV than the previous generation UCLA Miniscope V3 and 12-fold increase in FOV than the current generation UCLA Miniscope V4 (movie S1), we expect that this imaging platform will generate qualitatively and quantitatively new data for neuroscience applications in freely behaving large animals. As larger animals, rats for example, can often perform more sophisticated behaviors than mice (*41*), increasing the yield of neurons recorded simultaneously in these animals has the potential to yield novel discoveries about brain function, particularly when activity is encoded within a subset of the population (*42*–*44*). The MiniLFOV is fully open source, building off the already widely adopted open-source UCLA Miniscope ecosystem, and has been developed to reduce the technical and economic hurdles common in novel tool adoption, making it broadly accessible to the global neuroscience community.

## RESULTS

### System design

**Fig. 1.**
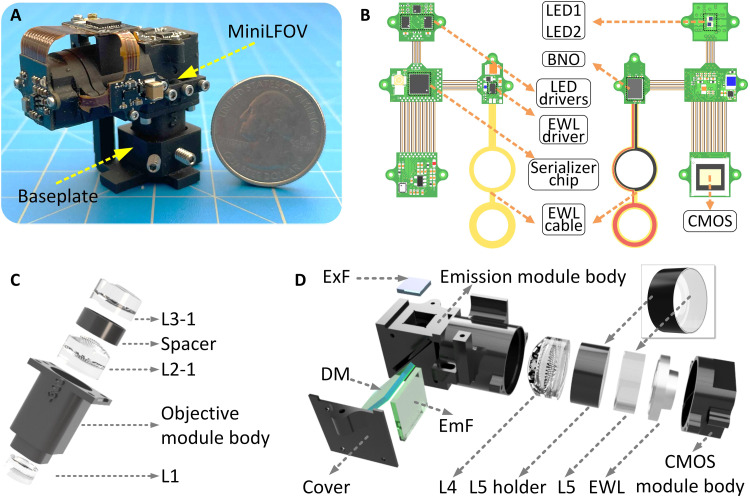
Design of MiniLFOV. (**A**) Photograph of MiniLFOV with baseplate. (**B**) Rigid-flex PCB of the MiniLFOV consisting of four rigid PCBs connected by an internal flex printed circuit. Two LEDs are housed on the LED circuit board and driven by two led drivers with an I^2^C digital potentiometer for brightness control. An EWL driver and an EWL cable holding the EWL are housed alongside an absolute orientation sensor (BNO055) for collecting head orientation data. A 5-MP monochromatic CMOS image sensor (MT9P031) is used for capturing Ca^2+^ fluorescence and sending digitized image data to the serializer system. A serializer chip serializes the imaging data and sends it over a coaxial connector to communicate with a custom Miniscope DAQ system. (**C**) Objective module of the MiniLFOV (1.8-mm-WD configuration) containing a three-dimensional (3D) printed objective module body, three achromatic lenses (L1, L2-1, and L3-1), and a spacer between L2-1 and L3-1. (**D**) Emission and sensor module of the MiniLFOV. The emission module consists of a 3D-printed emission module body with a 3D-printed cover, aspheric lens L4, a plane concave lens L5, L5 holder, excitation filter (ExF), dichroic mirror (DM), and emission filter (EmF). The sensor module consists of a 3D CMOS mole body holding the EWL and a mounting pocket for the CMOS image sensor.

The MiniLFOV (Fig. 1A and tables S1 and S2) consists of three modules, an objective module, an emission module, and a sensor module, holding the optical components and custom rigid-flex printed circuit board (PCB). The PCB consists of an excitation light-emitting diode (LED) subcircuit, an EWL tuning and head orientation subcircuit, a CMOS image sensor subcircuit, and a power-over-coax and serializer subcircuit, all interconnected by an embedded flex printed circuit (Fig. 1, A and B). The 1.8-mm-WD objective module (Fig. 1C) contains three achromatic lenses (#45-345, #49-656, #45-092, Edmund Optics) and a three-dimensional (3D) printed spacer. The emission module (Fig. 1D) holds the excitation filter (ET470/40x, Chroma), dichroic mirror (T500spxr, Chroma), emission filter (ET525/50m, Chroma), aspherical lens (#49-658, Edmund Optics), 3D-printed lens holder, and concave lens (#45-019, Edmund Optics). The sensor module is designed for holding the EWL driven by an EWL driver (MAX14515, Maxim) and a 5-MP monochrome CMOS image sensor (MT9P031, Onsemi) (see Fig. 1B). The main modules are screwed together with M1 thread-forming screws and 0-80 socket head screws.

Power, communication, and image data are packed into a single, flexible 50-ohm coaxial cable (CW2040-3650SR, Cooner Wire) using power-over-coax filtering and a serializer/deserializer pair for bidirectional control communication and unidirectional high-bandwidth data streaming (TI DS90UB913A/DS90UB914A).

The MiniLFOV interfaces with the open-source Miniscope DAQ software to stream, visualize, and record neural dynamics and behavioral data. The software enables multiple miniscope and behavioral camera video streams, allowing for multi-animal neural and behavioral recordings. This DAQ platform allows for the adjustment of excitation intensity, EWL focus, image sensor gain, and frame rate. The DAQ supports synchronizing with external devices through an outputted frame synchronization signal or an input trigger to externally trigger the recording. The list of the components used is given in table s3.

MiniLFOV accessories also include a reinforced baseplate attached to the skull with dental/bone cement, which allows consistent mounting of the MiniLFOV and a protective cap to cover the GRIN lenses implanted. Details of assembling the MiniLFOV are given in Materials and Methods and fig. S1.

### Optical performance

**Fig. 2.**
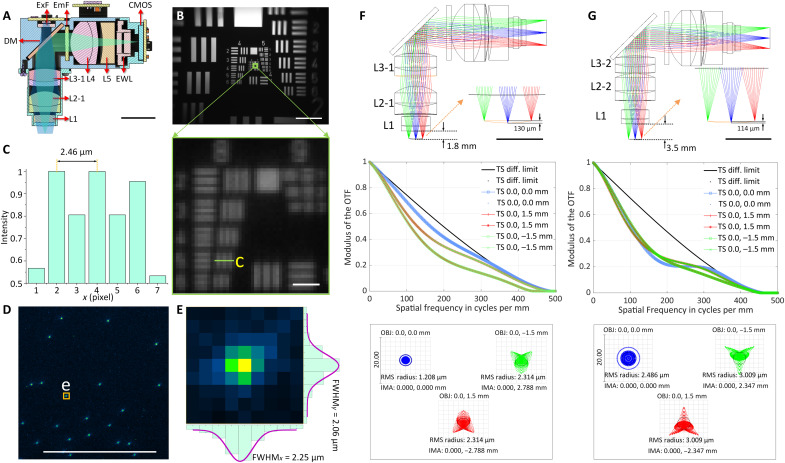
Optical design and performance of the MiniLFOV. (**A**) Cross section of the MiniLFOV assembly, including an objective module (1.8-mm WD) with three achromatic lenses (L1, L2-1, and L3-1), an emission module with one achromatic lens (L4), one concave lens (L5), and one EWL. This includes an excitation filter (ExF), dichroic mirror (DM), and emission filter (EmF). Scale bar, 10 mm. (**B**) FOV (3.1 mm × 2.3 mm) shown using the Negative USAF 1951 Hi-Resolution Target (#55-622, Edmund Optics). Scale bars, 500 μm (top) and 10 μm (bottom). (**C**) The value 2.5 μm (group 8 element 5, green box; 406 lps/mm) can be resolved from the green line in (B) (bottom). (**D**) Fluorescent beads (1 μm) imaged with the MiniLFOV. Scale bar, 250 μm. (**E**) *xy* sections of one selected bead marked in (D) with FWHM*_x_*= 2.25 μm and FWHM*_y_*=2.06 μm, respectively. (**F**) Zemax simulation of emission path shows the 1.85-mm-WD configuration with 130-μm field cuvature in the object space (top), MTFs on the image plane (middle), and spot diagram of the optics (bottom). In the spot diagram, root mean square (RMS) radius is 1.208 μm at the center and 2.134 μm at (0, 1.5 mm/−1.5 mm). The magnification of the optics is given by 1.86 calculated from the spot diagram. (**G**) Zemax simulation of emission path shows the 3.5-mm-WD configuration with 114-μm field curvature in the object space (top), MTFs on the image plane (middle), and diagram (bottom). A 3.5-mm WD (right) has RMS radius of 2.486 μm at the center and 3.009 μm at (0, −1.5 mm/1.5 mm), with a magnification of 1.56 and a 3.6 mm × 2.7 mm FOV achievable experimentally.

The optics of the system were designed and optimized using Zemax OpticsStudio. The excitation path (blue in Fig. 2A) and the emission path (green in Fig. 2A) are split by the dichroic mirror, which also folds the emission path to lower the center of mass of the system (Fig. 2, A, F, and G). The emission filter is placed in the emission path to block possible excitation light leakage and backscatter. The high resolution of the system and large FOV are achieved by using five off-the-shelf lenses with optimized distance and sequence between them. The 1.8-mm-WD configuration of the system is designed to have a 0.25 numerical aperture (NA) at the object space, with the modulation transfer function (MTF) curves reaching zero at 500 lps/mm (2 μm) on the image plane. The field curvature of the optics in objective space across a 3-mm FOV is 130 μm, and the magnification of the system is 1.9 (Fig. 2F). The optical system also enables an electrically adjustable WD of ±100 μm in the object space with the EWL (Fig. 1D and figs. S2 and S3). To collect the large FOV at its highest resolution, a CMOS image sensor is used with 2592 pixels × 1944 pixels of 2.2-μm pixel size, with a 3.1 mm × 2.3 mm FOV achieved (Fig. 2B and fig. S2). In practice, the achieved spatial resolution of the system is 2.5 μm (Fig. 2, B and C) at the center of the FOV, dropping to 4.4 μm at the edge. The resolution is also validated with full width at half maximum (FWHM) values of 1-μm beads imaged (Fig. 2, D and E, and figs. S3 and S5). Driven with a 66.667-MHz pixel clock, the sensor runs at 11 frames per second (fps) at full resolution and 23 fps with 2× horizontal and vertical binning. With 2× pixel binning, 4.4-μm resolution is achieved at the center of the FOV (dropping to 6.2 μm at the edge) and is the configuration used for all data collection shown here. For higher frames per second (up to 15 fps at full resolution and 30 fps at 2× binned resolution), a 96-MHz clock can be used but requires shorter or larger diameter coaxial cables for stable use. Higher frames per second can additionally be achieved by cropping the recording window or increasing the binning factor. To optically excite Ca^2+^ indicators across its large FOV, two LEDs (LXZ1-PB01 and LUXEON) are available in the excitation path with a band-pass excitation filter placed below to narrow their spectral band. With 2 pixel × 2 pixel binning and maximum analog gain (MiniLFOV_bin2x; gain = 8×), the MiniLFOV has twice the sensitivity than that of a Miniscope V4 at maximum analog gain (gain = 3.5×), resulting in a 2× decrease in excitation power needed per unit area. A comparison of the sensitivity and signal-to-noise ratio (SNR) of Miniscope V3, Miniscope V4, and MiniLFOV is shown in fig. S4. We have also validated that there is no difference in SNR for regions of interest in the center of FOV and the periphery testing with Negative USAF 1951 Hi-Resolution Target without GRIN lens inserted between the MiniLFOV and the Target.

The 3.5-mm-WD configuration (Fig. 2G) uses L1, L2-2 (#49-657, Edmund Optics), and L3-2 (#45264, Edmund Optics) in the objective module and is designed to give additional space below the MiniLFOV for imaging through cranial windows or other thick samples. MTF curves reach zero at 450 lps/mm (2.2 μm) at the image plane, and the root mean square radius is 2.5 μm on-axis (*x*, *y* = 0 mm) and 3.0 μm off-axis (*x* = 0, *y* = 1.5 mm; *x* = 0, *y* = −1.5 mm). In practice, the achievable resolution is 3.5 μm, or 362 lps/mm, at the center of the FOV with full resolution at 11 fps. A resolution of 6.2 μm is achievable with 2× horizontal and vertical binning to run at an acquisition rate of 23 fps. The magnification is ×1.56, resulting in a full FOV of 3.6 mm × 2.7 mm.

### Imaging place cells in hippocampal CA1

Studies of the neural representations of space have been prominent ever since the discovery of place cells in the 1970s (*45*). Many pyramidal neurons within the dorsal hippocampus of rats respond when the animal occupies a particular location within an environment (*46*). Neurons with such a spatial response are called “place cells,” and it is believed that the hippocampus constructs an internal map of an environment (a “cognitive map”), which is crucial for spatial navigation and memory (*11*). Because of the laminar nature of the CA1 pyramidal layer and relative ease of access using implanted optical probes, many experiments on spatial navigation and episodic memories have begun using miniaturized endoscopy in this brain region (*11*, *17*). However, most experiments have been restricted to mice. Here, we performed Ca^2+^ imaging in the hippocampal dorsal CA1 region of rats to demonstrate the feasibility and extended capabilities of MiniLFOV recordings in freely behaving larger animal models.

Currently available open-source UCLA Miniscopes support imaging up to around a 1-mm^2^ FOV, which is sufficient to yield hundreds of imaged neurons in the mouse hippocampus (*19*). However, in animal models that are larger than mice (such as rats), a larger FOV is often needed to achieve similar cell counts due to the slightly larger size of cell bodies (*14*, *15*, *47*). Furthermore, Ca^2+^ imaging in larger animals can pose additional difficulties: Imaging through thicker tissue layers (such as through the stratum oriens to the pyramidal layer in CA1) causes greater tissue scattering, and GCaMP expression can be suboptimal using commercially available viral vectors that are often optimized for use in mice. A high-sensitivity, MiniLFOV system is needed to ameliorate these problems.

**Fig. 3.**
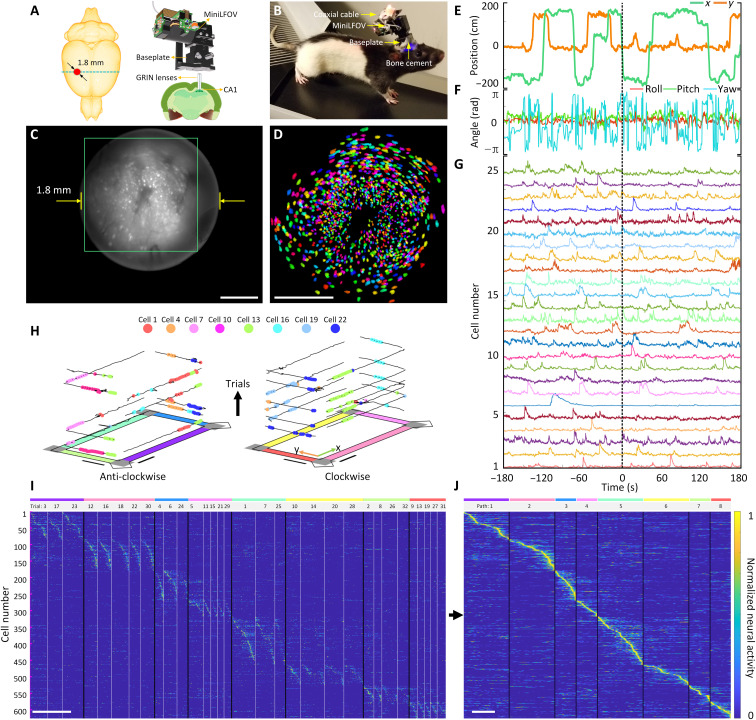
Strategy for imaging hippocampal dorsal CA1 in freely behaving rats. (**A**) Schematic of the imaging area in the rat brain. A custom relay GRIN lens is implanted above CA1 to relay fluorescence to the intermediate image plane formed between the top of the GRIN lens and bottom of the MiniLFOV. (**B**) Rat with MiniLFOV mounted on the skull via the reinforced MiniLFOV baseplate. (**C**) Maximum projection from a 15-min single recording session after motion correction. Scale bar, 500 μm. (**D**) Pixel area outlined in the green box in (C) showing extracted contours of cells (*N* = 1357) from CNMF-E analysis via CaImAn, colored randomly. Scale bar, 500 μm. (**E**) Example *x* and *y* track position as the rat runs in the 2D environment. (**F**) Example head orientation in terms of the roll, pitch, and yaw Euler angles. (**G**) Example extracted Ca^2+^ traces with CaImAn pipeline for 25 cells recorded during the session after low-pass filtering. (**H**) Deconvolved neural activity of eight example place cells spatially plotted on top of traversals along the track divided up by trial number and direction. Scale bars, 50 cm. (**I**) Normalized deconvolved neural activity of 626 place cells across all arm traversals sorted by the location of peak spatial activity rate shown in (J). Data shown here are speed thresholded (20 cm/s) to remove bouts of stationary active along traversals. Scale bar, 10 s. (**J**) Normalized spatial neural activity rate along all paths for these 626 place cells. Scale bar, 125 cm.

To record place cell activity from the dorsal CA1 region in rats, we imaged Ca^2+^ dynamics in GCaMP7s-expressing neurons in dorsal CA1 (Fig. 3A and figs. S6, S8, and S11), while rats performed navigation of a rectangular track environment. An overview of installing and removing the MiniLFOV is shown in figs. S6 and S7. At around a total mass of 20 g (which includes the MiniLFOV, baseplate, and cement), rats can easily wear the whole device without any signs of difficulty when running (Fig. 3B). In total, imaging data from 15 rats across months of recordings were recorded with the MiniLFOV in this task, but we present one exemplary session of one rat to demonstrate the effectiveness of the system. Example histology from 15 rats, showing GCaMP expression with the pyramidal layer for CA1 beneath the implanted lens location, is in fig. S8. The average running speed along the center of the maze and their reward rate (rewards per minute) in the session is presented to show that the MiniLFOV does not restrict the freely moving of the mice (fig. S9). Figure 3C shows a maximum projection image from an example 15-min motion-corrected video recording captured by the MiniLFOV. The raw video is recorded at 23 fps and around 15 min with 972 pixels × 1296 pixels for each frame after within-sensor 2× pixel binning and then further cropped to the pixel region containing the relay GRIN lens (720 pixels × 720 pixels). In this example recording, a total number of 1357 cells can be detected and extracted using extended constrained nonnegative matrix factorization (CNMF-E) analysis via calcium imaging data analysis (CaImAn) (Fig. 3D) (*29*, *30*). Recordings took place on a rectangular track (2.5 m × 1.25 m; fig. S10), with two reward feeders on two corners of the track. Positions on the track (Fig. 3E) and head orientation (Fig. 3F) of the rat were extracted from a software synchronized behavioral camera and on-board head orientation sensor, respectively (see Materials and Methods and fig. S10). Low pass–filtered Ca^2+^ traces of 25 example cells are shown in Fig. 3G.

As expected, a substantial rat, head or population of the cells (626; 46.2%) is spatially tuned along at least one direction on the four arms on the rectangular track. A cell is considered a place cell if it has significantly higher spatial information than chance (spatial information above at least 95% of 500 randomly shifted deconvolved neural activity for each cell) (*48*). The behavior of the rat was serialized into eight paths (four arms with two running directions) (Fig. 3, H to J). Place cells recorded during the session were modulated by both position and direction of travel and span across all regions of the track (Fig. 3, I to J). An example video showing the position of the ientation, and place cell activity during behavior is given in movies S2 and S3. These data demonstrate how the MiniLFOV can improve experimental efficacy by yielding more cells in larger research models, broadening the horizon of potential hypotheses for researchers. Demonstration of the MiniLFOV’s capability in terms of consistently recording activity from the same population of cells across multiple sessions is shown in fig. S11. The feasibility and capability of the MiniLFOV imaging cortical activity in the rat across multiple sessions are also validated with an example freely moving recording in a circular open field (80 cm diameter) while imaging Ca^2+^ dynamics of neurons in the anterior cingulate cortex (ACC). Viral expression of GCaMP8m was mediated by 1.2-μl injection of AAV9-Syn-GCaMP8m (#162375, Addgene), and neuronal activity was recorded through an implanted 1-mm-diameter GRIN lens from Thorlabs (G1P11, Thorlabs), shown in fig. S12 and movie S4. A 1-mm lens was used because of the proximity to the midline sinus, which makes implanting larger lenses more difficult, improving the success of the surgery and reducing potential overlap with the more lateral motor cortex.

### Optional wire-free recording in rats

**Fig. 4.**
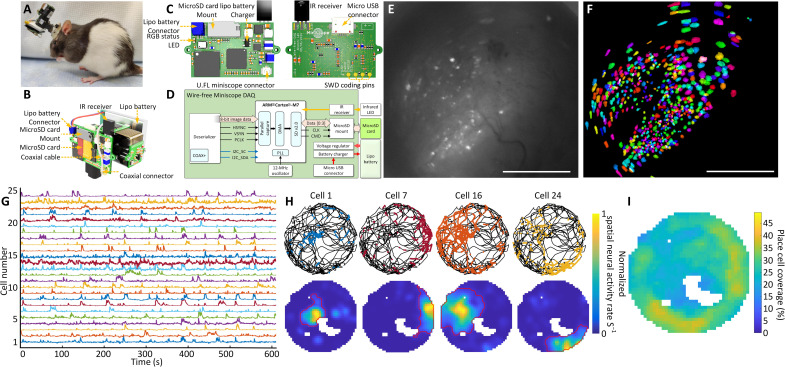
Wire-free imaging of hippocampal CA1 neurons in freely behaving rats in a circular open field. (**A**) Photograph of a rat wearing MiniLFOV with wire-free DAQ and single-cell lithium-polymer battery. (**B**) Three-dimensional rendering of the MiniLFOV equipped with wire-free DAQ and single-cell lithium-polymer battery. (**C**) Top and bottom PCB rendered layouts. (**D**) Block schematic of wire-free DAQ. (**E**) Maximum projection from a 14-min recording session after motion correction. Scale bar, 500 μm. (**F**) Contours of extracted cells (*N* = 575) from CNMF-E analysis via CaImAn, colored randomly. Scale bar, 500 μm. (**G**) Low pass–filtered Ca^2+^ transients from 25 example cells from (F). (**H**) Examples of spatially modulated neural activity for four place cells during exploration in the open field. Top: Speed thresholded spatial location of deconvolved neural activity (colored dots) superimposed over rat’s trajectory (black line). Bottom: Binned spatial neural activity rate maps for the same example cells. Red contour denotes the spatial outline of a place field based on a greater than 5% threshold of the binned spatial neural activity rate. (**I**) Combined open-field place field coverage from all 438 detected place cells (76%) showing full coverage of the environment with overall increased coverage near edges.

The MiniLFOV can record neural activity without the constraint of being tethered to DAQ hardware mounted off the animal. By mounting a novel wire-free DAQ (3.5 g) system to the side of the MiniLFOV or on a backpack and connecting it to the MiniLFOV through a 4-cm-long, 50-ohm coaxial cable, any wired UCLA Miniscope can operate in a wire-free configuration (Fig. 4, A and B). A single-cell 400-mAh lithium-polymer battery (7.5 g) is used to power the wire-free DAQ and MiniLFOV (supports close to 1 hour of continuous recording), and a microSD card (Class 10 UHS-I microSD, Kingston) is used for on-board data and configuration storage (Fig. 4, B to D). Once the wire-free DAQ is powered on, an on-board microcontroller unit (MCU) (ATSAME70N21A, Microchip) reads configuration data from the microSD card and then implements that configuration in the MiniLFOV. Configuration parameters include excitation intensity, EWL focus, and frame rate, gain, and FOV window of the image sensor. A status LED is integrated onto the wire-free DAQ for displaying current device state and visually synchronizing recording with behavior cameras (Fig. 4C). The wire-free DAQ uses an IR remote control receiver to receive digital commands, encoded into a 38-kHz IR carrier frequency, from an IR transmitter to trigger recording remotely. This implementation of IR communication allows for one-way, wireless data transfer to the wire-free DAQ and MiniLFOV. During recording, data from the attached MiniLFOV are streamed into the memory of the MCU by a direct memory access channel. Each acquired frame is timestamped and then saved onto the microSD card. The DAQ rate of the wire-free DAQ is limited to a pixel clock rate of around 24 MHz by the on-board MCU’s parallel capture mode. In addition, the MCU can maintain a maximum of ~11-Mbps write speed to the microSD card, placing additional constraints on the rate of image acquisition and storage. Because of these constraints, we chose the recording window size to be 608 pixels × 608 pixels at 15 fps (although it can achieve 20 fps), corresponding to 1.4 mm × 1.4 mm FOV in object space (relay GRIN lens is 1.8 mm in diameter). In the configuration used, around 5.5 MB is written to the SD card per second, allowing a 64-GB microSD card to support multihour recording.

The feasibility and capability of the wire-free MiniLFOV configuration are validated with an example rat freely moving in a circular open field (80 cm diameter) as unrestricted behavior and Ca^2+^ dynamics in GCaMP7s-expressing neurons in dorsal CA1 are recorded simultaneously. The maximum projection of a motion-corrected 14-min recording session is shown in Fig. 4E with a total of 575 extracted cells (Fig. 4F). Low pass–filtered Ca^2+^ traces from 25 example cells are shown in Fig. 4G. A large majority of cells (*N* = 438; 76%) satisfied our place cell criteria with spatial information above at least 95% of 500 circularly shuffled sessions. Four example place cells show the rat’s trajectory (black lines) and spatial location of deconvolved neural activity (colored dots) (Fig. 4H, top), showing clear place preference of neural activity. These example cells’ spatial neural activity rate maps are shown in Fig. 4H. The red outlined contour denotes the edge of a place field calculated by detecting a 5% cutoff of the binned spatial neural activity rate surrounding the place field. The combined open-field place field coverage from all 438 detected place cells shows the full coverage of the circular open field with overall increased coverage near the edges (Fig. 4I). This wire-free configuration enables untethered behavior during neural imaging in larger animals and has the potential to improve naturalistic behavior by removing cable torque and looming cues. In addition, the wire-free DAQ extends the capabilities of the UCLA Miniscope to simultaneous multi-animal recordings and experiments where tethering is infeasible.

### Imaging cortex in head-fixed mice

**Fig. 5.**
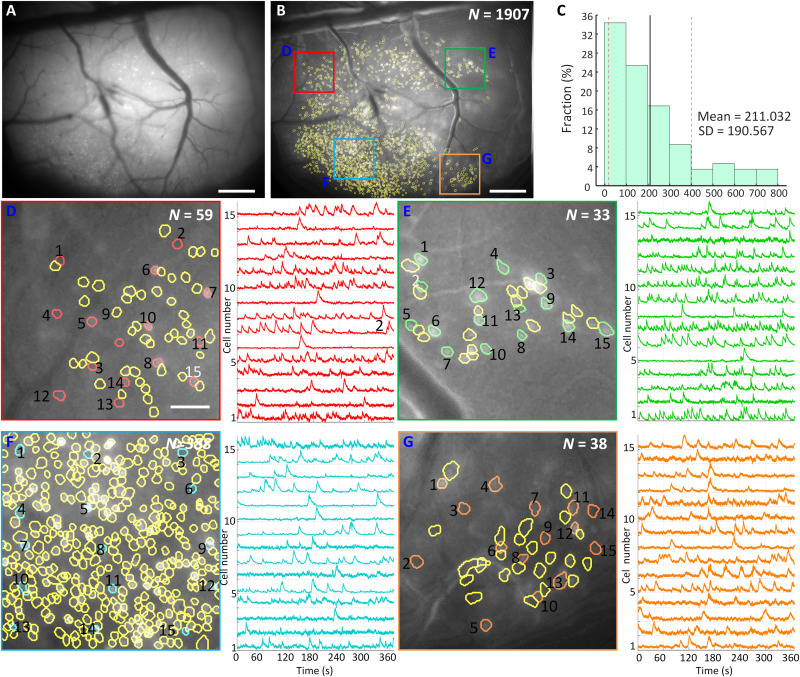
Ca2^+^ imaging of dorsal cortex through a 4 mm × 4 mm cranial window in head-fixed mice. (**A**) Maximum projection from a 7-min recording session after motion correction. Scale bar, 500 μm. (**B**) Maximum intensity projection image of the raw video after motion correction and background removed with contours of 1907 extracted cells from CNMF-E analysis via CaImAn circled in yellow. The colored boxes indicate four subregions that are zoomed in and shown in (D) to (F). Scale bar, 500 μm. (**C**) Distribution of cell numbers inside 500 randomly chosen 1-mm-diameter circular regions (FOV of Miniscope V4) in the entire FOV of the MiniLFOV. The mean cell number is 211.032, and the SD is 190.567. (**D**) Map of 59 cells in the red-boxed area in (B) and 15 randomly chosen cells with their low pass–filtered Ca^2+^ transients for the numbered cells are shown in red. Scale bar, 100 μm. (**E**) Map of 33 cells in the green-boxed area in (B) and 15 randomly chosen cells with their low pass–filtered Ca^2+^ transients for the numbered cells are shown in green. (**F**) Map of 388 cells in the cyan-boxed area in (B) and 15 randomly chosen cells with their low pass–filtered Ca^2+^ transients for the numbered cells are shown in red. (**G**) Map of 38 cells in the orange-boxed area in (B) and 15 randomly chosen cells with their low pass–filtered Ca^2+^ transients for the numbered cells are shown in cyan.

With single-cell resolution across the entire 3.6 mm × 2.7 mm FOV (3.5-mm-WD configuration; see Fig. 2G), the MiniLFOV is capable of large-scale imaging of cortical ensembles in head-fixed mice, freely moving rats, and larger animals. Here, we demonstrate that the MiniLFOV enables single-cell resolution across the entire FOV (Fig. 5A) by imaging GCaMP6f-expressing dorsal cortex (*49*, *50*) through a 4 mm × 4 mm cranial window in head-fixed mice running on a 29-mm circular treadmill (Fig. 5A and fig. S13A). Initial attempts at cortical imaging through a coverslip in the rat cortex were unsuccessful at resolving single-cell activity, with only large out-of-focus background activity visible. Cellular activity (<200 μm) should be able to be resolved, but layer 2/3 neurons in the rat cortex are too deep for coverslip imaging. Research restrictions implemented at the onset of the coronavirus disease 2019 (COVID-19) pandemic prevented further attempts, so we instead used an already existing surgical preparation of mouse cortical imaging. This enabled us to demonstrate the efficacy of the entire FOV.

A MiniCAM (fig. S13A), an open-source behavior camera developed by the UCLA Miniscope group, was used to record the movement of the mouse (fig. S13B). Maximum intensity projection image of the raw video after motion correction with background removed is shown in Fig. 5B. The bright white spots in the image are putative cells. Contours of 1907 extracted cells from CNMF-E analysis via CaImAn circled in yellow distributed across the full FOV. To compare the number of cells Miniscope V4 and MiniLFOV can capture, we randomly chose 500 1-mm-diameter circular regions, which is the FOV of Miniscope V4, to calculate the distribution of the cell number. The mean cell number is 211.032 and the SD is 190.567 (Fig. 5C). As a comparison, MiniLFOV captured nine times the number of cells on average. To show that the MiniLFOV can resolve single cells, four subregions located near the four corners in the entire FOV are chosen and magnified in Fig. 5 (D to G), and the background is removed to highlight the putative cells [bright white spots in Fig. 5 (D to G)] and the contours of the extracted cells (circled in yellow). In each subregion, 15 cells are randomly chosen with extracted Ca^2+^ traces plotted on the right to show the visual assessment of the SNR during the session after low-pass filtering. Both the contours and the corresponding Ca^2+^ traces have validated the performance of MiniLFOV with respect to single-cell resolution. Similar results from a second mouse are shown in fig. S14.

## DISCUSSION

We present a large FOV open-source miniature microscope platform aimed at extending the capabilities of microendoscopic imaging approaches in large rodents and nonhuman primates, such as rats, marmosets, and macaque monkeys. We have presented both table-top and in vivo validation of imaging in two animal models (rats and mice) in various surgical preparations (1.8-mm GRIN, 1-mm GRIN, and head-fixed coverslip) and in various brain regions (hippocampus, ACC, and retrosplenial cortex). The system can image up to a 3.6 mm × 2.7 mm FOV at 23 fps with single-cell resolution, has an electrically adjustable WD of up to 3.5 mm ± 100 μm, incorporates an absolute head orientation sensor, and weighs under 14 g, which is under the weight of many head-mounted neural recording devices for rats like microdrives. The MiniLFOV achieves a 20-fold increase in sensitivity compared to the previous generation Miniscope V3, and a 2-fold increase in sensitivity compared to the current generation mouse Miniscope V4. The MiniLFOV includes a modular objective lens configuration, enabling multiple imaging approaches for deep and superficial brain imaging. The MiniLFOV can be easily attached to and removed from a chronically implanted baseplate that has been designed to provide rigid mechanical support for stable recording in freely behaving animals larger than a mouse. The baseplate mounting mechanism also provides an accurate and repeatable mounting to image the same neural population and track individual neurons across recording sessions (figs. S6, S7, and S11).

The MiniLFOV provides a flexible and cost-effective imaging system for rats, which are currently an underrepresented animal model in most calcium imaging studies. The MiniLFOV has been validated in freely behaving rats by imaging neurons in the dorsal CA1 layer of the hippocampus and in the ACC. To date, only a small handful of papers have demonstrated calcium imaging in rats, and here, we have demonstrated a system capable of the highest yield of CA1 cells within a single recording. Users also have the option of imaging through a cranial window or using thinner relay GRIN lenses for deeper brain imaging, all of which are compatible with the MiniLFOV. Previous recordings of freely behaving rats using original Miniscope V3 designed for mice were limited compared to mouse studies, typically yielding around 100 to 300 cells per recording (*44*, *51*). With the presented MiniLFOV, we can now achieve simultaneous recordings of more than 1000 neurons in freely behaving rats during a single 15-min session (Fig. 3). When imaging across the entire FOV of the MiniLFOV, close to 2000 active neurons could be imaged across the dorsal cortex in head-fixed mice (Fig. 5). This platform greatly extends Ca^2+^ imaging capabilities in freely behaving larger animal models and opens new avenues of research that were previously limited by low simultaneous cell counts. Imaging in larger animal models also reduces the proportional damage caused by aspiration or implantation procedures.

The entire open-source UCLA Miniscope platform has also been extended for wire-free operation. This has the potential to reduce behavior interference (such as cable tangling and unintentional looming cues) as well as enable recordings from multiple animals socially interacting (*31*) and animals exploring large environments. Performance and capabilities of three configurations of the MiniLFOV, i.e., 1.8-mm-WD, 3.5-mm-WD, and wire-free, are listed in table S1. Furthermore, with independently controlled dual-LED excitation, the MiniLFOV can support future two-color excitation configurations by changing one of the LEDs as well as changing filters and dichroic mirror (*52*). With a mass of only 13.9 g, it is feasible for two MiniLFOVs to be mounted onto the skull of a rat for multiregion imaging. The MiniLFOV broadens the application of Ca^2+^ imaging to larger, freely behaving animals than previously realized, enabling large-scale and high cell count recordings in both cortical and deep brain structures. Moreover, the MiniLFOV, wire-free DAQ, and UCLA Miniscope online resources provide an open-source, accessible, and cost-effective option for neuroscientists seeking to adopt Ca^2+^ imaging into their laboratories.

## MATERIALS AND METHODS

### MiniLFOV design, manufacturing, and assembly

#### Design

length = 14 mm; #49-658, Edmund Optics) and a concave lens (*d* = 12 mm, focal length = −48 mm; #45-019, Edmund Optics) with a lens. The optical system of the MiniLFOV was designed using Zemax OpticsStudio. The goal of the optical system is to produce a miniature microscope with large FOV and high NA, in which the desired NA is set to be 0.25 in the objective space and FOV to be a minimum of 3 mm. Lenses are chosen to achieve the optical performance while maintaining a compactable and lightweight size. The distance between lenses is optimized to achieve single-cell resolution across the entire FOV with minimal field curvature. Three achromatic lenses L1 (*d* = 6.25 mm, focal length = 60 mm; #45-345, Edmund Optics), L2-1 (*d* = 9 mm, focal length = 12 mm; #49-656, Edmund Optics), and L3-1 (*d* = 9 mm, focal length = 27 mm; #45-092, Edmund Optics) are used to build the 1.8-mm-WD objective module with a 3.5-mm-tall spacer placed between L2-1 and L3-1. L1 (*d* = 6.25 mm, focal length = 60 mm; #45-345, Edmund Optics), L2-2 (*d* = 9 mm, focal length = 18 mm; #49-657, Edmund Optics), and L3-2 (*d* = 9 mm, focal length = 36 mm; #45264, Edmund Optics) are chosen to build the 3.5-mm-WD objective module (Fig. 2G). One aspherical lens (*d* = 12.5 mm, focal holder are placed into the emission module body to form the tube lens of the MiniLFOV. The EWL (Corning Arctic 58 N, Varioptics/Corning) is placed into the sensor module body (Fig. 1D and fig. S1) for electronic focus adjustment. The MiniLFOV module bodies (objective module body, emission module body, and sensor module body), filter cover, baseplate, and protective cap are designed in Autodesk Fusion 360 (educational license) and printed with black resin (FLGPBK04, Formlabs) by a table-top stereolithographic 3D printer (Form 3, Formlabs) to produce a lightweight, compact, and low-cost assembly. Three filter slots are designed on the emission module body for mounting a custom-diced excitation filter (4 × 4 × 1.1 mm, ET470/40x, Chroma), a dichroic filter (14 × 10 × 1 mm, T500spxr, Chroma), and an emission filter (10 × 10 × 1 mm, ET525/50 m, Chroma). All the optical components can be easily assembled without the need for any epoxy or optical glue. A list of the optical components used is given in table S3. The circuit schematic and rigid-flex PCB layout are designed using KiCad, a free software suite for electronic design automation. The PCB is divided up into four rigid subcircuits, which include an excitation LED circuit, an EWL tuning and head orientation circuit, a CMOS image sensor circuit, and a power-over-coax and serializer circuit. The modularity of the PCB design enables quick modification or redesign of individual subcircuits without the need for modifying the entire PCB layout. The four subcircuits are connected by a double-sided embedded flex printed circuit (Fig. 1, A and B). The assembled objective module is attached to the emission module body with five 18-8 Stainless Steel Socket Head Screws (92196A052, McMaster-Carr). The emission module, sensor module, cover, and rigid-flex PCB are fastened together with M1 thread-forming screws (96817a704, McMaster-Carr) (see fig. S1). The open-source behavior camera (MiniCAM) for behavior tracking is developed by UCLA Miniscope group (https://github.com/Aharoni-Lab/MiniCAM) and compatible with the open-source UCLA Miniscope DAQ hardware and software. The MiniCAM consists of an M12 optical lens mount, a custom PCB housing a CMOS image sensor and supporting electronics, LED illumination ring with 16 red LEDs, and a 3D-printed case. The brightness of the LEDs can be adjusted through software for the optimal illumination in dark environments. The MiniCAM runs at around 50 fps with 1024 × 768 resolution and saves the behavioral data in AVI file format using an MJPG compression video codec directly supported within the UCLA Miniscope software.

#### Wiring

A single flexible coaxial cable (50 ohms; CW2040-3650SR, Cooner Wire) is used for power, communication, and image data transmission relying on a passive power-over-coax filter and a serializer/deserializer pair (TI DS90UB913A/DS90UB914A) for combining and separating dc power, low-speed bidirectional communication, and high-speed unidirectional imaging data. For coaxial cable lengths longer than 2.5 m, an external 6-V power supply should be connected to the UCLA Miniscope DAQ, replacing the power supplied by the USB connection to the MiniLFOV. With a total cable diameter down to 0.3 mm (100065-0023, Molex) and compatibility with active and passive, low-torque commutators, this design minimizes the impact of cabling on animal behavior. The DAQ hardware and software are based on the UCLA Miniscope project’s previous work (http://miniscope.org/index.php/Main_Page), with updated firmware (https://github.com/Aharoni-Lab/Miniscope-DAQ-Cypress-firmware) and software (https://github.com/Aharoni-Lab/Miniscope-DAQ-QT-Software) to enable video streaming and controlling of the MiniLFOV. This updated software enables excitation LED brightness adjustment, focus adjustment by EWL, real-time ∆*F*/*F* and fluorescent trace visualization, frame rate selection, and gain adjustment and supports real-time pose estimation using DeepLabCut-Live (*53*) through embedded Python. A list of the hardware and software used is given in table S3.

### Animals

All experimental protocols were approved by the Chancellor’s Animal Research Committee of the University of California, Los Angeles, in accordance with the U.S. National Institutes of Health (NIH) guidelines. All animals and procedures for the anterior cingulate imaging were conducted under the New York University Animal Welfare Committee protocol 2021-1146 in accordance with the U.S. NIH guidelines.

### Surgical implantation

#### Rat hippocampal imaging

Three-month-old Long-Evans rats (Charles River Laboratories) underwent two survival surgeries before behavior training to record fluorescent Ca^2+^ activity from hippocampal CA1 cells. During the first surgery, rats were anesthetized with 5% isoflurane at 2.5 liters/min of oxygen and then maintained at 2 to 2.5% isoflurane, while a craniotomy was made above the dorsal hippocampus. Next, 1.2 μl of AAV9-Syn-GCamp7s (Addgene) was injected just below the pyramidal layer [−3.6 anterior-posterior (AP), 2.5 medial-lateral (ML), 2.6 dorsal-ventral (DV)] via a 10-μl NanoFil syringe (World Precision Instruments) mounted in a Quintessential Stereotaxic Injector (Stoelting) controlled by a Motorized Lab Standard Stereotax (Harvard Apparatus). The left or right hemisphere was balanced across all animals. One week later, the rat was again induced under anesthesia and four skull screws were implanted to provide stable mounting for the GRIN lens implant and MiniLFOV baseplate. The craniotomy was reopened to a diameter of 1.8 mm, and cortical tissue and corpus callosal fibers above the hippocampus were aspirated away using blunted 27- and 30-gauge needles. Following this aspiration, and assuring no bleeding persisted in the craniotomy, a 1.8-mm-diameter GRIN lens (#64-519, Edmund Optics) was implanted over the hippocampus and cemented in place with methacrylate bone cement (Simplex-P, Stryker Orthopedics). The dorsal surface of the skull and the bone screws was cemented with the GRIN lens to ensure stability of the implant, while the surface of the lens was left exposed. Two to 3 weeks later, rats were again placed under anesthesia to cement a 3D-printed baseplate above the lens. First, a second GRIN lens was optically glued (Norland Optical Adhesive 68, Edmund Optics) to the surface of the implanted lens and cured with ultraviolet light. The pitch of each GRIN lens was approximately 0.25, so combining 2 in series provided roughly a 0.5 pitch. This half pitch provides the translation of the image at the bottom surface of the lenses to the top while maintaining the focal point below the lens, effectively becoming a relay GRIN lens. This relay implant enables access to tissue deep below the skull surface. It has been simulated that the aberrations of the 1.8-mm GRIN near the edges generate a rotational stretching of the neural footprints, which can be alleviated with high-quality GRIN lenses (provide roughly a 90% clear aperture) or commercially available lower NA GRIN lenses with near-full FOV image achievable. Cannula (2.5 to 3 mm in diameter) was also reported to be used for imaging hippocampal CA1 instead of GRIN lens (*54*–*57*). The MiniLFOV was placed securely in the baseplate and then mounted to the stereotax to visualize the Ca^2+^ fluorescence and tissue. The baseplate was then cemented in place above the relay lenses at the proper focal plane, the MiniLFOV was removed from the baseplate, and cement allowed to cure. Once rats had been baseplated, they were placed on food restriction to reach a goal weight of 85% ad lib weight and then began behavioral training. Briefly, the MiniLFOV was mounted into a baseplate with two set screws (4-40 thread × 1/4″ long) and then positioned above the implanted lenses while the baseplate was cemented in place with bone cement (Fig. 3B). The baseplate body was 3D printed (20 mm × 20 mm outer dimensions) with two set screws on the side (Fig. 1A and figs. S6 and S7) for rigidly mounting a protective cap when not recording (protecting the GRIN lens and surgical region) and MiniLFOV body during experiments.

#### Rat histology

At the end of the experiment, rats were anesthetized with isoflurane, intraperitoneally injected with 1 ml of pentobarbital, and then transcardially perfused with 100 ml of 0.01 M phosphate-buffered saline (PBS) followed by 200 ml of 4% paraformaldehyde in 0.01 M PBS to fix the brain tissue. Brains were sectioned at 40-μm thickness on a cryostat (Leica), mounted on slides, and then imaged on a confocal microscope (Zeiss) to confirm green fluorescent protein (GFP) expression and GRIN lens placement.

#### Cortical imaging of rat ACC

Three-month-old rats (Charles River Laboratories) were anesthetized and mounted in the stereotax as described for hippocampal imaging and following the previously described methods (*42*). A small 1-mm hole was made above the dorsal ACC region (+2.0 mm AP, 0.7 mm ML), and a 0.6-μl bolus of AAV9-Syn-GCaMP8m was injected at both 0.9 and 1.7 mm below the skull surface over 10 min each. The injection needle was kept in place after the second (more dorsal) injection for 20 min before removal. Implantation of the GRIN lens then took place in the same surgery. First, a 0.9-mm drill burr was slowly lowered to 1.8 mm below the skull surface, and then a 1-mm GRIN lens (G1P11, Thorlabs) was implanted at 2.0 mm ventral to the skull surface. Subsequent surgical procedures follow those described above. Three weeks later, rats were baseplated as described in the hippocampal imaging, except without the addition of a 0.25-pitch additional lens. Perfusion and histology procedures follow those described above.

#### Cranial window imaging on head-fixed mice

Adult C57BL/6N Tac (3 to 5 months old) male mice were singly housed on a 12-hour light/dark cycle. Mice were bilaterally microinjected with 500 nl of AAV1.Syn.GCaMP6f.WPRE.SV40 virus (100837, purchased from Addgene) at 20 to 120 nl/min into the dorsal cortex using the stereotactic coordinates: −1.7 and −2.3 mm posterior to bregma, 0.5 mm lateral to midline, and −0.8 mm ventral to the skull surface. Mice underwent window implantation surgeries as previously described (*58*). Briefly, a square region of skull ~4 mm in width was marked using stereotactic coordinates (center at bregma −2.2 mm AP). The skull was thinned using a dental drill and removed. After cleaning the surgical site with saline, a custom cut sterilized coverslip (square, 4 mm × 4 mm) was placed on the dural surface and fastened with adhesive and dental acrylics to expose a square window of approximately 3.5 mm spanning the midline. Three weeks later, a small 3D-printed baseplate was cemented onto the animal’s head atop the previously placed dental cement. In the second mouse, an aluminum bar with two threaded holes was attached to stabilize the mice during imaging sessions. Following baseplating or attachment of headbar, mice underwent handling (3 days) and habituation (3 days) to acclimate them to the treadmill and head fixation.

### Rat behavioral training

#### Ca^2+^ imaging in freely behaving rats

After baseplating, rats were given 15-min sessions every 48 hours to perform a linear alternation task on a rectangular linear maze where one arm (2.5 m) directly connects the reward locations, while the other three arms in total are indirect and, combined, twice as long (5 m). These arms form a 2.5 m × 1.25 m rectangle with the reward locations at the corners of one long side, elevated 1 m off the ground. The paths are 10-cm wide spanned by small metal bars along the entirety of the short and long paths. The reward zones are coated wood, 14 cm wide, 20 cm long, at a 45° angle with the two paths. The entire maze has a short 1-cm wall to provide a safe graspable ledge in case the rat loses its footing. The outer perimeter of the short path has a slightly taller wall angled 45° away from the path to protect the wiring connected to the short path bars. Twenty-milligram chocolate sucrose pellets are delivered through a metal tube at the end of the reward zone via an automated hopper, controlled by a computer running Cheetah software (Neuralynx) feeding position information to custom MATLAB scripts, which controlled pellet delivery as the rat ran from one reward zone to the other. Rewards are delivered at the opposite reward location from their previous reward as the rat passes through the center of either path. Both paths yield delivery of two pellets, and there is no time-out period after delivery. The rat must enter the most recent reward delivery location before the opposite side will be rewarded, forcing an alternation behavior. The rats are allowed to choose either path, and since they are equally rewarded, they demonstrate a preference for the short path (2.5-m arm) over the long path (the other three arms) after only a few sessions. Rats are given 15-min recording sessions every 48 hours to minimize potential photobleaching of the Ca^2+^ indicator. Once a rat demonstrated a consistent short path preference and sufficient running behavior (>2 short paths per minute and a 2:1 short:long ratio in the first 10 min for two consecutive sessions), we introduced a manipulation in the subsequent session. Effects of these manipulations are omitted in this article, and we only evaluate period of consistent behavioral performance in absence of induced learning.

#### ACC and wire-free imaging on freely behaving rats

For testing the functionality of the wire-free configuration, the experiment was conducted by putting the rat into a circular open-field (80 cm diameter) to record the unconstrained exploration of the environment and the neural activity simultaneously. An additional red LED is attached onto the MiniLFOV body for the behavior camera to track its position. Animals were food-deprived up to 85% of their original weight after which recordings commenced. Sucrose grain pellets (20 mg) were thrown in the enclosed environment every 20 s at random locations within the open field, keeping the animal in continuous locomotion, thereby allowing close to complete sampling of the environment (*59*).

### Data analysis

#### Behavior video analysis

For experiments involving freely behaving rats, videos of behaviors were captured in AVI format using an overhead camera (30 fps; Logitech C270). The position of the rat is extracted by tracking the position of the red LED attached to the top of the MiniLFOV recorded by a behavioral camera. The pixel location of the LED is found by detecting the highest pixel value region within the grayscale behavior recording frames, a correction of the optical aberration of the camera lens is applied, and then the pixel value location is converted to real-world coordinates. For the head-fixed experiment in mice, a MiniCAM (fig. S13A) was used for recording movement speed of the mice. The movement of the mice was extracted from the recorded motion of a circular treadmill (22.9 cm diameter) marked with 1-cm wide alternating dark and white bars on the surface (fig. S13B).

#### Head orientation analysis

The quaternion values (*q_w_*, *q_x_*, *q_y_*, *q_z_*), read from the on-board head orientation sensor (BNO055, Bosch) following each frame acquisition, were saved in CSV file format during recording. These data were converted to Euler angles (roll, pitch, yaw) offline for further analysis, with the matrix used below

$$R=\left[\begin{array}{ccc} r_{11} & r_{12} & r_{13}\\ r_{21} & r_{22} & r_{23}\\ r_{31} & r_{32} & r_{33} \end{array}\right]=\left[\begin{array}{ccc} 1-2{q_{y}}^{2}-2{q_{z}}^{2} & 2q_{x}q_{y}+2q_{w}q_{z} & 2q_{x}q_{z}-2q_{w}q_{y}\\ 2q_{x}q_{y}-2q_{w}q_{z} & 1-2{q_{x}}^{2}-2{q_{z}}^{2} & 2q_{y}q_{z}+2q_{w}q_{x}\\ 2q_{x}q_{z}+2q_{w}q_{y} & 2q_{y}q_{z}-2q_{w}q_{x} & 1-2{q_{x}}^{2}-2{q_{y}}^{2} \end{array}\right]$$

$$\mathrm{roll}=\arctan\;\left(\frac{r_{23}}{r_{33}}\right),\mathrm{pitch}=\arctan\;\left(\frac{\sqrt{{r_{11}}^{2}+{r_{12}}^{2}}}{-r_{13}}\right),\mathrm{and}\;\mathrm{yaw}=\arctan\;\left(\frac{\sin(\mathrm{roll})\times r_{31}-\cos(\mathrm{roll})\times r_{21}}{\cos(\mathrm{roll})\times r_{22}-\sin(\mathrm{roll})\times r_{32}}\right)$$

Further correction of the Euler angles depends on the axis orientation of the head orientation sensor and the mounting orientation of the MiniLFOV during experiments.

#### MiniLFOV data preprocessing

The imaging data from the CMOS image sensor are saved in AVI file format using an uncompressed (GREY) video codec directly supported within the Miniscope DAQ software, and all the recorded videos for each animal in each session were concatenated into one single video file and cropped to the region of interest pixel region using custom Python 3 scripts before further processing.

#### Wired experiment on rats in rectangular linear maze

Image stacks of Ca^2+^ dynamics (972 pixels × 1296 pixels) were cropped to the pixel region containing the relay lens stack (720 pixels × 720 pixels). We next used the CaImAn Python processing pipeline to perform nonrigid motion correction followed by image source extraction using constrained nonnegative matrix factorization for endoscopic recordings (CNMF-E) (*29*, *30*) to identify and extract the spatial contours and fluorescent Ca^2+^ activity of individual neurons. Fast nonnegative deconvolution OASIS (https://github.com/zhoupc/OASIS_matlab) was used to deconvolve the slow time course inherent in the GCaMP fluorophore (*30*) to estimate underlying neural activity. The resulting deconvolved Ca^2+^ activity can be interpreted as the sum of neural activity within each frame scaled by an unknown constant, and we refer to this measure as the “temporal neural activity” of a cell. Because the temporal neural activity of each cell is scaled by an unknown number that varies across cells, we normalized each cell’s temporal neural activity. Spatial neural activity rates were calculated path by path (paths 1 to 8) using 2-cm-wide spatial bins and a speed threshold of greater than 20 cm/s. Temporal neural activity and occupancy of the animal were spatially binned and then smoothed using a Gaussian kernel with σ = 5 cm. Then, the binned neural activity was divided by the binned occupancy to calculate the spatial neural activity rate of each cell.

The information content of the spatial neural activity rate map (in bits) (*19*) of a single neuron was defined as

$$I=\sum_{i=1}^{N}\;p_{i}\frac{\lambda_{i}}{\bar{\lambda }}\log_{2}\frac{\lambda_{i}}{\bar{\lambda }},p_{i}=\frac{t_{i}}{\sum_{i=1}^{N}t_{i}},\bar{\lambda }=\sum_{i=1}^{N}p_{i}\lambda_{i}$$

where *t_i_* represents the occupancy time spent in the *i*th bin of total *N* bins and λ*_i_* represents the neural activity rate in the *i*th bin. $\bar{\lambda }$ is the mean neural activity rate. The significance of the spatial information was calculated using two circular shuffling procedures. For each shuffling procedure, the random permutations were generated by frame-shifting the speed-thresholded sequence of temporal neural activity for each trial (total, 32 trials) by a random interval between 0 and the number of frames for each trial with frame number out of range being circularly wrapped to the beginning (*60*). Then, random permutations were generated by frame-shifting the entire sequence of temporal neural activity along the rat’s eight paths by a random interval between 0 and the number of frames, with frame number out of range being circularly wrapped to the beginning. This procedure was repeated 500 times with random shifts to determine a significance measure for the spatial activity rate map of each neuron. The significance measure is the percentile of the true information content value within the distribution of the information content values from the 500 randomly shuffled sessions. Cells with information content significantly above chance (*P ≥* 0.95) based on the circularly shuffled distribution are labeled place cells.

#### Head-fixed experiment imaging dorsal cortex in mice

As described earlier, Ca^2+^ recordings were processed as to yield the spatial contours of neurons and their deconvolved Ca^2+^ activity. Distribution of cell numbers is calculated inside 500 randomly chosen 1-mm-diameter circular regions (FOV of Miniscope V4) in the entire FOV of the MiniLFOV.

#### Wire-free imaging on freely behaving rats

Wire-free MiniLFOV data were extracted from microSD cards and saved as uncompressed 8-bit AVI video files using custom Python code for processing and analysis. Ca^2+^ recordings were processed as described earlier to yield the spatial contours of neurons and their deconvolved Ca^2+^ activity. Spatial neural activity rates were calculated using 2 cm × 2 cm spatial bins and a speed threshold of greater than 5 cm/s. Temporal neural activity and occupancy of the animal were spatially binned and then smoothed using a Gaussian kernel with σ = 3 cm. A minimum occupancy threshold was set to be 100 ms where spatial bins that did not meet this threshold were excluded from all subsequent analysis. The binned neural activity was divided by the binned occupancy to calculate the spatial neural activity rate of each cell. Information content of the spatial neural activity rate map was calculated with the same equation as described in the wired experiment on rats in the rectangular track, and the significance of the spatial information content was calculated using a circular shuffling procedure. Random permutations were generated by time-shifting the entire sequence of positions along the rat’s path by a random interval between 10 s and the total recording minus 10 s, with the time-out of the range being wrapped to the beginning (*60*). The 10 s at the beginning and the end of the recoding is occluded to remove the period of putting the rats into and out of the open field. This procedure was repeated 500 times to determine a significance measure for the spatial activity rate map of each neuron. The significance measure is the percentile of the true information content value within the distribution of the information content values from the 500 randomly shuffled sessions. Cells with information content significantly above chance (*P* ≥ 0.95) based on the circularly shuffled distribution are labeled place cells. For cells defined as place cells, their place fields are defined as the region that (i) contains five adjacent bins with binned neural activity rate above 95% of that of all randomly shuffled binned neural activity rates and (ii) extends to all connected bins with a binned neural activity rate of at least 5% of the place field’s peak binned neural activity rat. This approach allows for a robust detection and spatial region definition for place cells with single as well as multiple place fields. The place field spatial region is shown as red contours in Fig. 4H.

### Matching cells across sessions

Contours computed from CaImAn were normalized by their own maximum value and then thresholded at 0.5 to generate compact contours for all cells. Artifact contours detected on the edge of the GRIN lens were manually removed; then, each session was manually aligned using the contours and vasculature simultaneously using custom MATLAB scripts. In addition to linear translations, it was sometimes necessary to radially scale these data as well as to account for slight differences induced by camera placement and different focal positions from the EWL. For all rats, we aligned and matched three consecutive recording sessions (48 hours apart) where running performance was stable. Contour alignment was performed using CellReg to register cells across all sessions of interest based on their centroid distances and spatial contour correlations (*61*). Signal scattering through issue and background noise inherent to single-photon miniature microscopy increases the difficulty of tracking cells across sessions; however, CellReg provides metrics for understanding the quality of identification based on multiple measures, allowing researchers to quantify their confidence of the registration. Distributions of centroid distances and spatial correlation for all cell pairs within 24 μm were computed, yielding a 2D distribution that can be modeled and given a probability threshold to match cell pairs. Cell pairs with a probability of >0.5 for both the centroid and correlation distributions were matched (typically centroid distances of <6 μm and spatial correlation of >0.8), which is expected when recording the same cell.

## Notes

View/request a protocol for this paper from *Bio-protocol*.
