# Supplementary material for: Miniscope-LFOV: A large-field-of-view, single-cell-resolution, miniature microscope for wired and wire-free imaging of neural dynamics in freely behaving animals
Source: Sci Adv. 2023 Apr 21;9(16):eadg3918. doi: 10.1126/sciadv.adg3918 (PMC10121160; doi:10.1126/sciadv.adg3918)
Supplement: Supplementary file 1 — Figs. S1 to S14 Tables S1 to S3 Legends for movies S1 to S4 [file sciadv.adg3918_sm.pdf]

Science Advances
AAAS

Supplementary Materials for

Miniscope-LFOV: A large-field-of-view, single-cell-resolution, miniature microscope for wired and wire-free imaging of neural dynamics in freely behaving animals

Changliang Guo *et al.*

Corresponding author: Daniel Aharoni, daharoni@mednet.ucla.edu

Sci. Adv. **9**, eadg3918 (2023)
DOI: 10.1126/sciadv.adg3918

The PDF file includes:

Figs. S1 to S14
Tables S1 to S3
Legends for movies S1 to S4

Other Supplementary Material for this manuscript includes the following:

Movies S1 to S4

Supplementary Materials

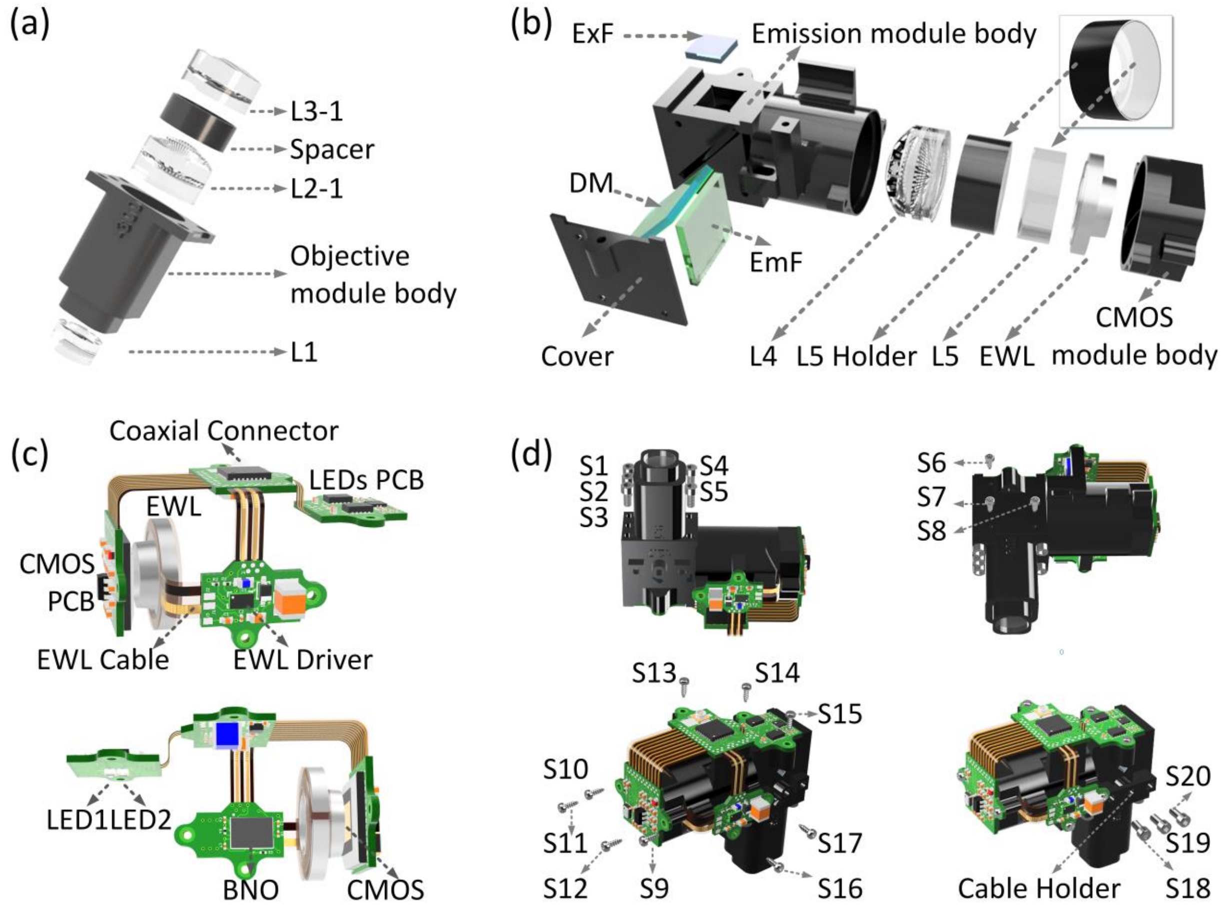

Fig. S1. Mechanical and optical assembly of MiniLFOV. The MiniLFOV consists of three main modules, objective module (details in a), emission module (details in b), sensor module, and a custom Rigid-Flex printed circuit board (PCB) (details in c), and they are screwed together with two types of screws (details in d). Objective module (1.8-mm WD) contains three achromatic lenses (L1, L2-1, and L3-1) with a spacer (3.5-mm tall) placed between L2-1 and L3-1, with a 3D printed objective module body to hold the components. 3.5-mm-WD objective module contains three achromatic lenses (L1, L2-2, and L3-2) with a spacer (2-mm tall) placed between L1 and L2-2. Emission module contains filters (excitation filter “ExF”, dichroic mirror “DM”, and emission filter “EmF”), and optics lenses (achromatic L4 and plane-concave lens L5), with a 3D printed emission module body to hold the filters and lenses. Sensor module holds the EWL with a 3D printed CMOS module body. The circuit module consists of 4 sub-circuits, an excitation LED circuit, an electrowetting lens tuning and head orientation circuit, a CMOS image sensor circuit for collecting Ca^{2+} imaging data, and a power-over-coax and serializer circuit (Fig. 1a, b) for supporting coaxial cable power and data transmission. Two LEDs are housed on the LED circuit board driven by two LED drivers with I^2C digital potentiometer for brightness adjustment. Electrowetting lens tuning and head orientation circuit consists of EWL driver, with EWL cable holding the EWL, to adjust the focus of the EWL and the absolute-orientation chip for collecting head orientation data. 5M CMOS sensor (MT9P031) is housed on the CMOS image sensor circuit for Ca^{2+} fluorescence image capturing. A serializer chip on the power-over-coax and serializer circuit serializes the imaging data and sends it over a coaxial connector to communicate with a

custom MiniscopeDAQ system. The objective module, emission module, sensor module and the circuits module are assembled by 12 (S6-S17) M1 thread-forming screws (96817a704, McMaster-Carr), and 8 (S1-S5, S18-S20) 18-8 stainless steel socket head screws (0-80 Thread Size, 92196A052, McMaster-Carr).

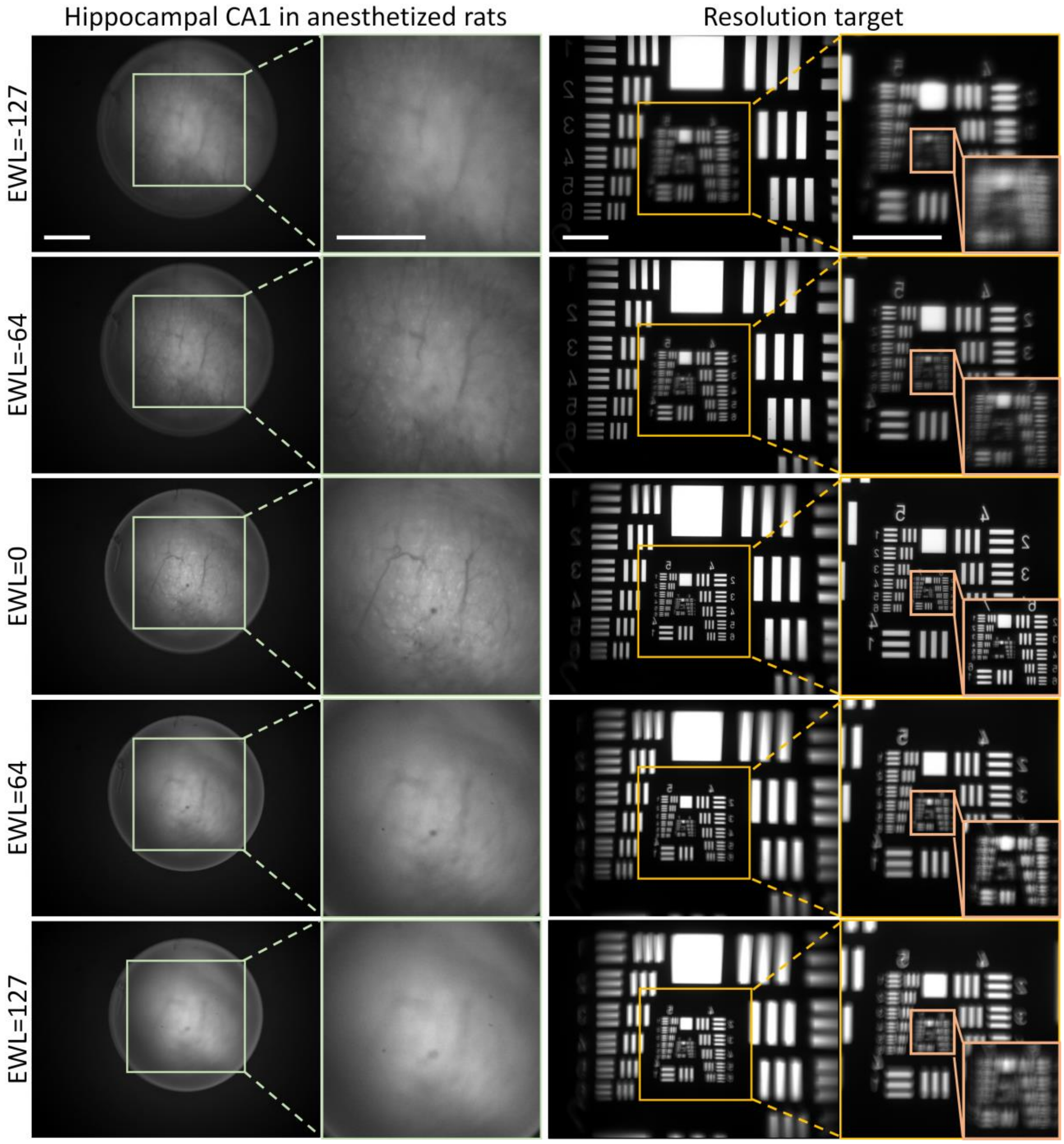

Fig. S2. Five imaging focal planes attained by changing the focal position with the electrowetting lens (EWL) in an anesthetized rat and Resolution target to show how the resolution changes. The EWL value is set to be -127, -64, 0, 64, and 127 respectively. Negative EWL values relate to the deeper/ventral focal plane and positive EWL value corresponds to more superficial/dorsal. The cells and blood vessels are in focus when EWL is 0. The imaging focal plane can be adjusted with the EWL, allowing researchers to compensate for slight displacements in MiniLFOV placement in the baseplate between sessions.

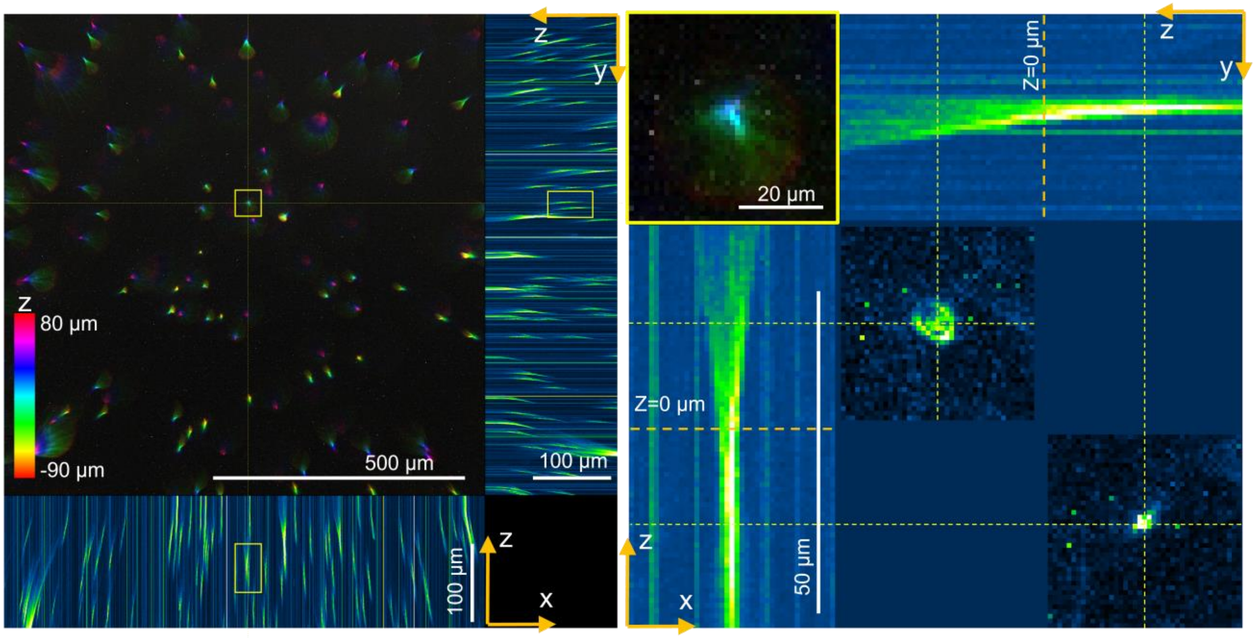

Fig. S3. Imaging of 1-μm fluorescent beads in 2% agarose gel by changing EWL lens from -90 μm to 80 μm to show how the imaging changes. 0.5 μL of 1-μm fluorescent beads (10 μL diluted into 990 μL normal saline) are mixed with 500 μL 2% agarose gel. 40 μL of the agarose containing the fluorescent beads was applied to a coverglass (0.14 -0.17mm, 20×20 mm) and sealed onto a coverslide (1 mm thick) with UV glue to generate thick agarose gel with the beads randomly distributed inside, and the specimen was ready for use after 10 min. The sample slide is then attached to a slide holder (with the coverglass facing up). The MiniLFOV is attached to a manual 3D translation stage and placed above the sample slide. The lateral position and height of the MiniLFOV was adjusted to optimize image quality for each imaging session. Only the center 800 × 800 pixel (0.957 mm × 0.957 mm) region is shown in this figure. The left figure shows the color coded volumetric beads by adjusting EWL from -90 μm to 80 μm (with total 221 z-slices taken), with their projected x−z and y−z views shown on the bottom and right. Negative EWL values relate to the deeper/ventral focal plane and positive EWL value corresponds to more superficial/dorsal. The tilting of the beads along z axis is caused by the changed magnification of the imaging when adjusting EWL values. Distortions can be seen when the EWL value is near the adjustment limit. An example fluorescent bead located near focal plane (EWL=0) is shown in the right figure with x-z and y-z projections shown.

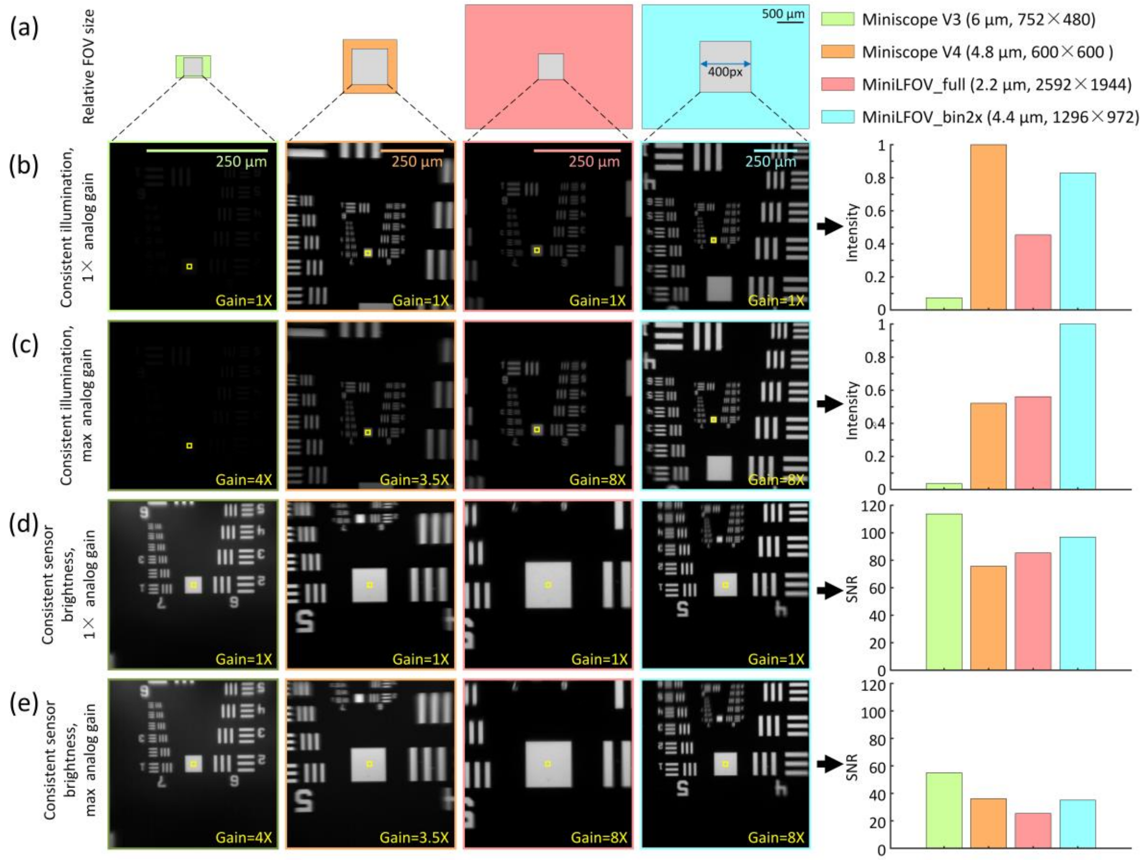

Fig. S4. Sensitivity and signal noise ratio (SNR) comparison of Miniscope V3, Miniscope V4, MiniLFOV full resolution (MiniLFOV_full), and MiniLFOV after 2×2 pixel binning (MiniLFOV_bin2x) using Resolution Target. A light source was placed under a Negative USAF 1951 Hi-Resolution Target (#55-622, Edmund Optics) with a thin diffuser placed between them to diffuse the light. Then a Miniscope V3, Miniscope V4, and MiniLFOV are attached to a 3D translation stage and placed above the target. The lateral position and height of the Miniscopes were adjusted to optimize image quality for each imaging session. A 30-second video was recorded with each Miniscope in cases of consistent illumination (1x, and max analog gain), and consistent sensor brightness (1x, and max analog gain). The sensitivity comparison of the Miniscopes was done by illuminating the back side of the Negative USAF 1951 Hi-Resolution Target with the light source remaining at a constant intensity. Sensor sensitivity was calculated as the mean pixel value within the 10 x 10 pixel box shown in yellow. The SNR comparison was done by adjusting the light source intensity to achieve equivalent pixel brightness (8bit gray scale value = ~200), across all Miniscopes in the chosen area on the sensors. Sensor SNR was calculated as the mean pixel value divided by the standard deviation of all pixels within the 10 x 10 pixel box shown in yellow. A 400 x 400 pixel mean image from each 30-second recording is shown. **(a)** Relative FOV size of Miniscope V3 (light green box, 650 µm x 420 µm), Miniscope V4 (orange box, 1-mm diameter), MiniLFOV (salmon, and cyan, 3.1 mm x 2.3 mm), and chosen 400 x 400 pixel area (gray box) in each case. Legend shows pixel size and full sensor resolution. **(b)** Sensitivity of Miniscopes with unity analog gain set for each. MiniLFOV is less sensitive than Miniscope V4 (83%). **(c)** Sensitivity of Miniscopes with maximum analog gain set for each. Higher sensitivity is achieved with MiniLFOV than Miniscope in this case (192%). **(d)** SNR of Miniscopes with unity analog gain set for each. **(e)** SNR of Miniscopes with maximum analog gain set for each.

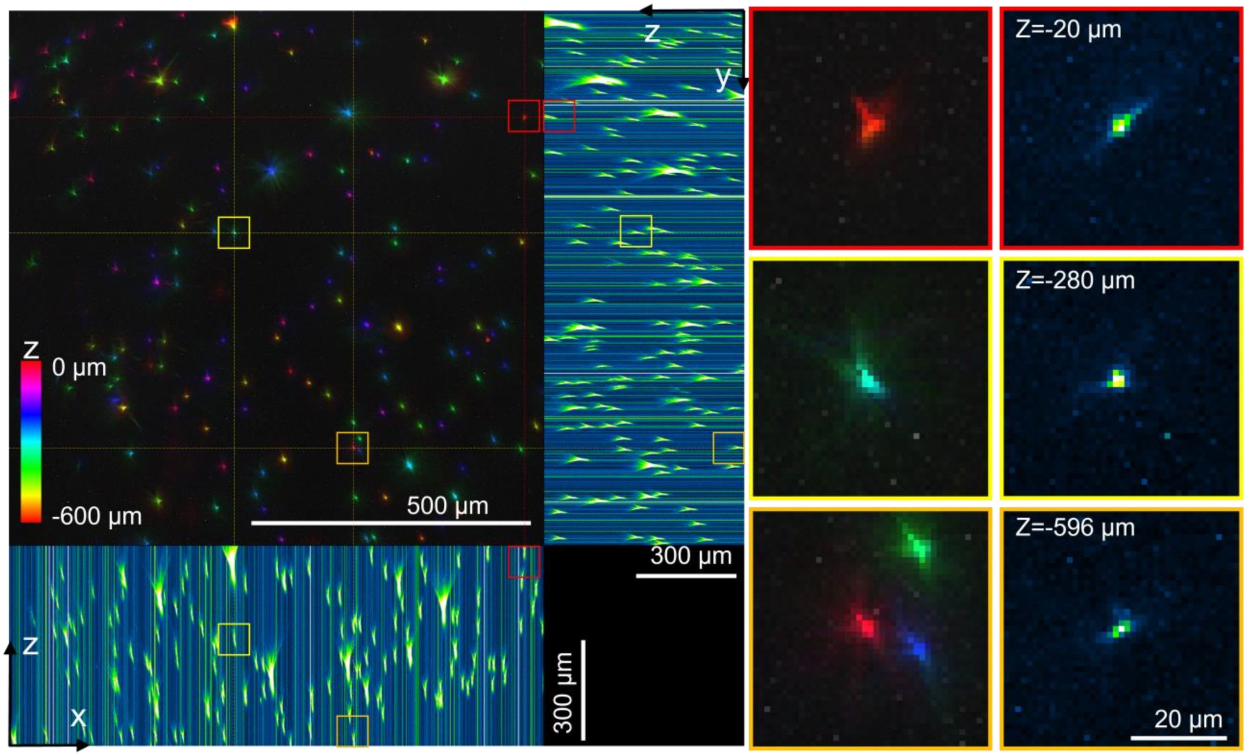

Fig. S5. Imaging of 1-μm fluorescent beads up to 600 μm deep into thick 2% agarose gel sample slide. 40 μL of the agarose containing the fluorescent beads was applied to a coverglass (0.14 -0.17mm, 20×20 mm) and sealed onto a coverslide (1 mm thick) with UV glue to generate thick agarose gel with the beads randomly distributed inside, and the specimen was ready for use after 10 min. The sample slide is then attached to a slide holder (with the coverglass facing up) mounted on a motorized z-axis linear translation stage, to adjust the z-axis position of the sample with up to 100 nm adjustment accuracy. Then the MiniLFOV is attached to a manual 3D translation stage and placed above the sample slide. The lateral position and height of the MiniLFOV was adjusted to optimize image quality for each imaging session. The left figure shows the imaged color-coded beads distributed in 3D space in agarose gel by adjusting the z-axis translation stage closer to the MiniLFOV from a start imaging depth 0 μm (the depth close to the MiniLFOV) to -600 μm deep into the agarose gel, to make the MiniLFOV focusing deeper into the agarose gel step by step, with total 301 images taken for the stack. Their projected x−z and y−z views are shown on the bottom and right. Only the center 800 × 800 pixel (0.957 mm × 0.957 mm) region is shown in this figure. 3 example beads located around -20 μm (red box), -280 μm (yellow box), and -596 μm (orange box) are shown in the right figures to validate the MiniLFOV can resolve the fluorescent beads at least down to 600 μm (see the bead inside the orange box) into the agarose gel. Bright straight lines in the x-z and y-z projection figures are noises from low excitation light intensity.

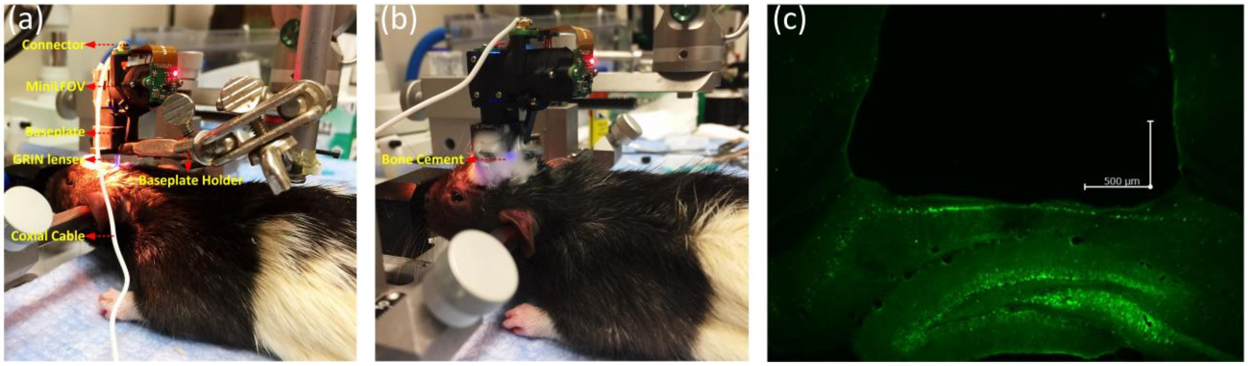

Fig. S6. MiniLFOV baseplating on the rat. (a) A second ~0.25-pitch GRIN lens is affixed atop of the first ~0.25-pitch GRIN lens (implanted during earlier surgery), to construct a relay optics setup (~0.5 pitch). Then the MiniLFOV baseplate is held by a stereotaxic clamp. The MiniLFOV, attached to the baseplate with two screws, is positioned above the second GRIN lens on an anesthetized ear-barred rat. The distance from the MiniLFOV to GRIN lens is adjusted to focus on the hippocampal CA1 region exhibiting the most active and in-focus neurons. (b) The MiniLFOV baseplate is then affixed to the animal via bone cement mixture. After baseplating, a protective cap is used to cover and protect the GRIN lens from dust and scratches. (c) Histological image of a brain slice using a confocal microscope (Zeiss) sectioned at 40-µm thickness on a cryostat (Leica) to confirm GFP and GRIN lens placement. Picture shows GFP expression in the dorsal hippocampus of the rat, approximately 2.6 mm below the skull surface; scale bar: 500 µm.

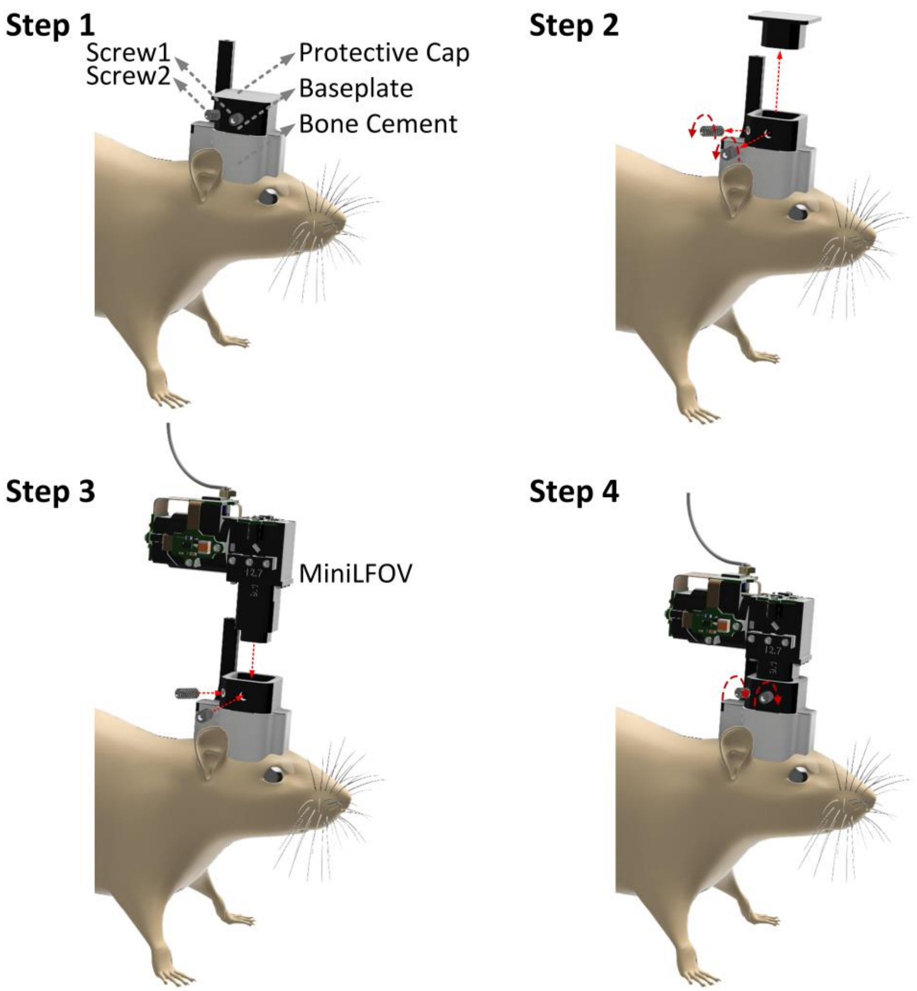

Fig. S7. Mounting the MiniLFOV on the rat's head. (Step 1) While the animal is in its home cage, a protective cap is used in the baseplate and secured with two set screws (4-40 x 1/4") to protect the GRIN lens from dust and scratches. At the start of the experiment, the protective cap is removed (Step 2) and the MiniLFOV is inserted into the baseplate and secured with two set screws (Step 3-4). At the end of the imaging session, the MiniLFOV is again replaced by the protective cap.

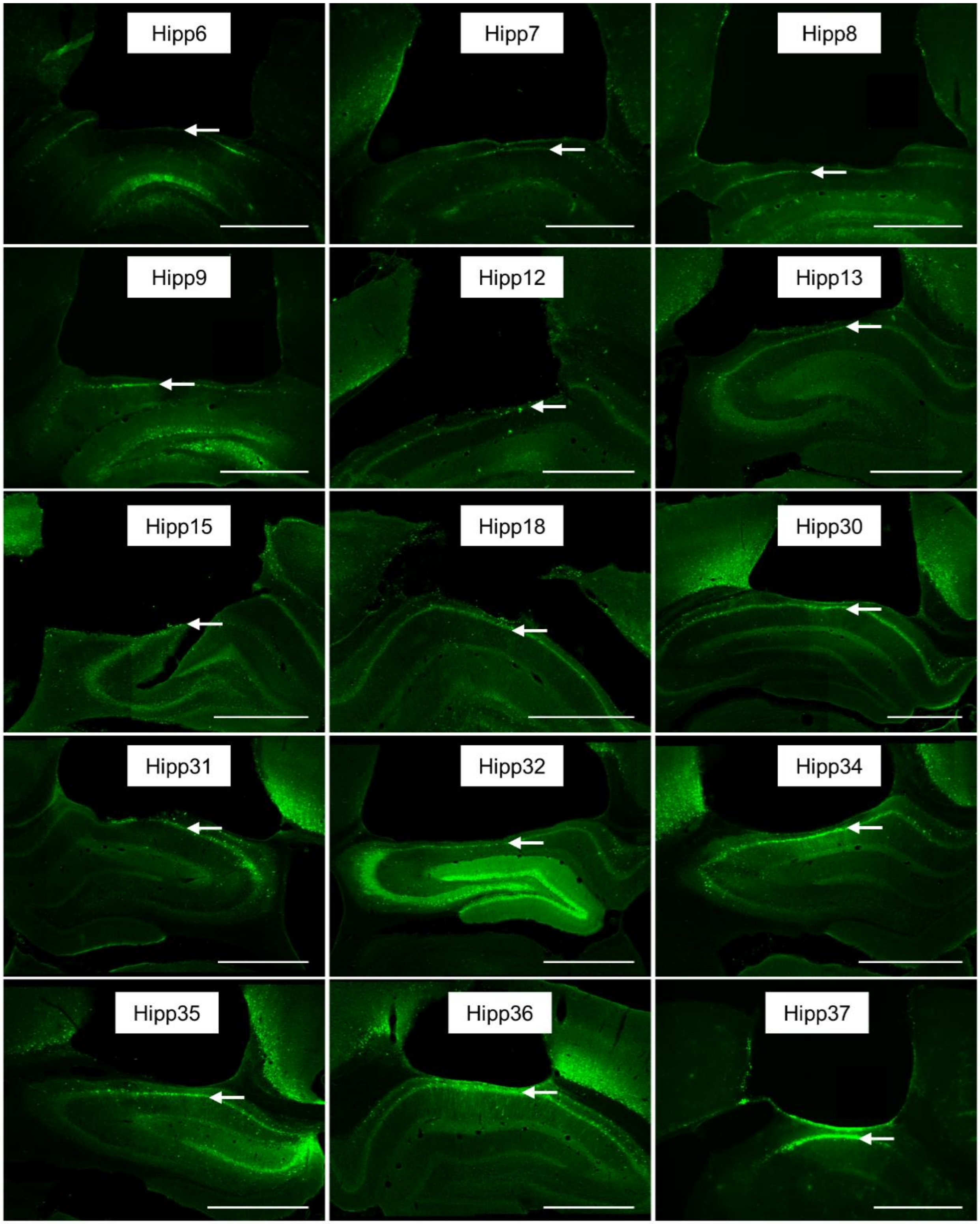

Fig. S8. Example histology from 15 rats, showing GCaMP expression with the pyramidal layer for CA1 beneath the implanted lens location (arrow). Scale bar: 1 mm.

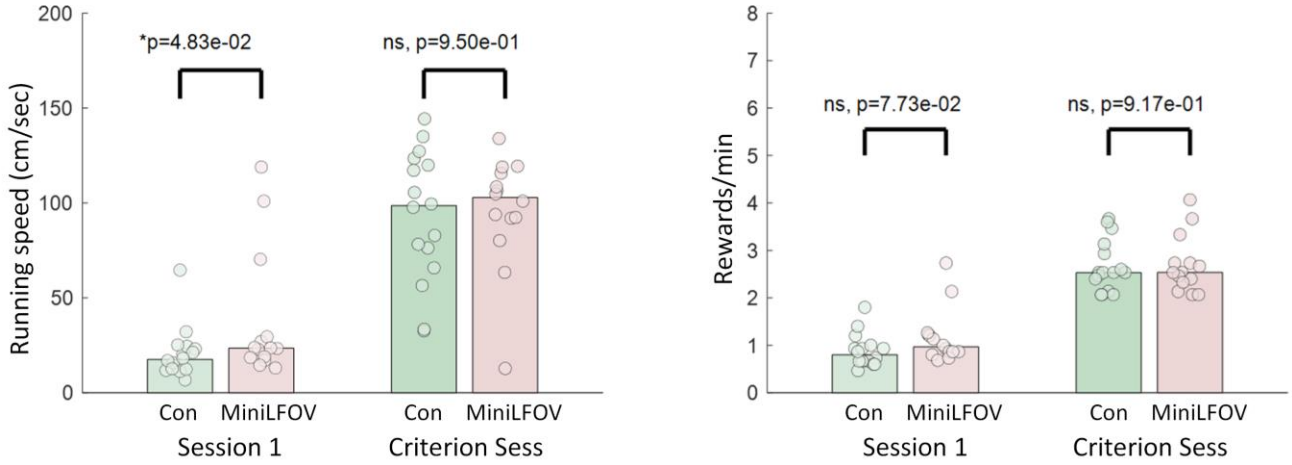

Fig. S9. The MiniLFOV does not impede behavior of ras in a linear alternation task. Rats implanted with the MiniLFOV (lens, baseplate, and camera, "MiniLFOV", *n*=14) are not significantly impaired in their running speed or reward retrieval rats during the first session or criterion session compared to unimplanted controls ("CON", *n*=16). It can be seen that although the rats with MiniLFOV mounted start out running a bit faster than controls, they are never impaired compared to controls in either measure, thus the MiniLFOV doesn't limit their movement ability.

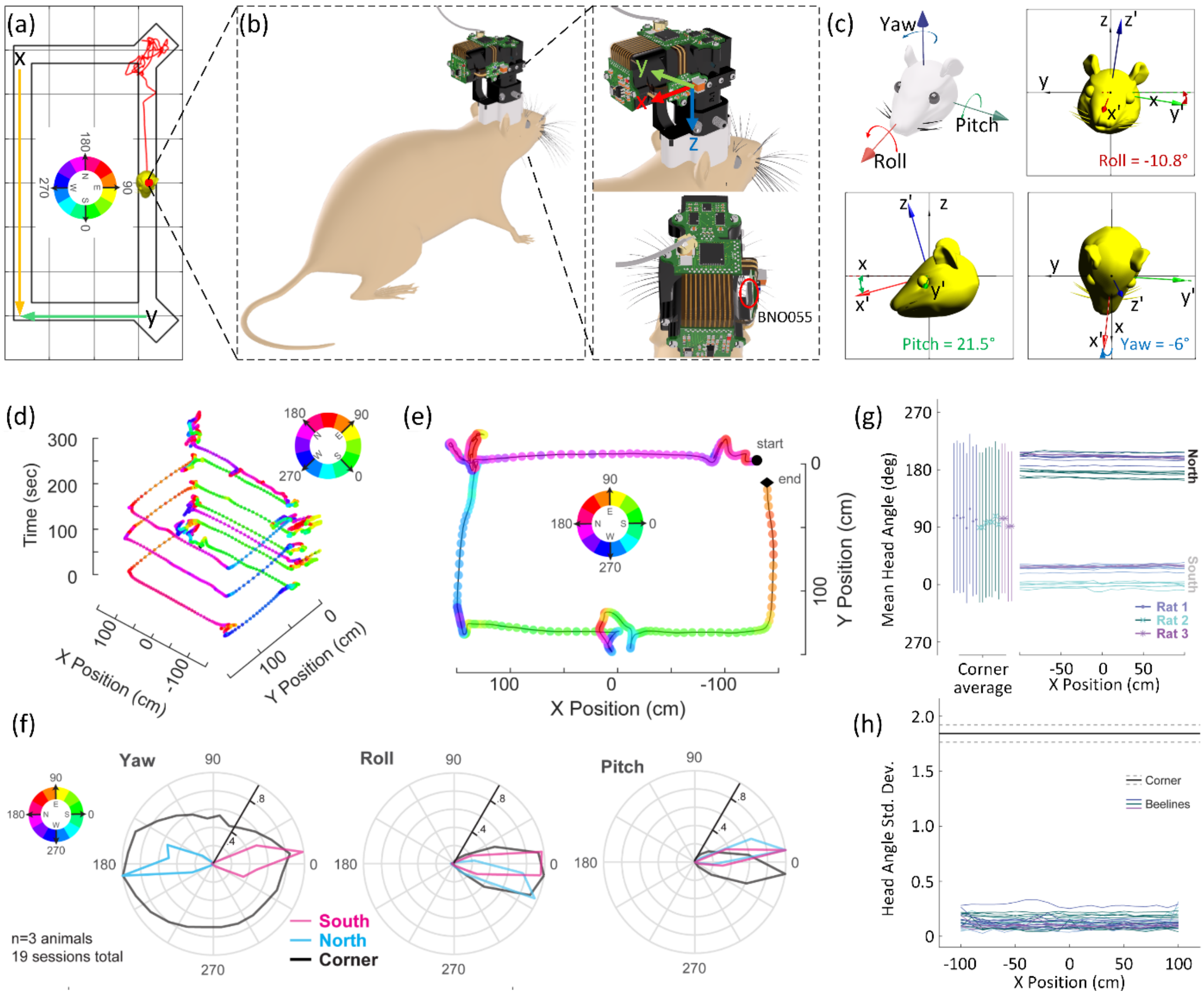

Fig. S10. Illustration of the head orientation data (roll, pitch, yaw) from head-orientation sensor (BNO055). (a) An example of a rat with the MiniLFOV mounted on its head running on a rectangular track. (b) 3D image showing the coordinate of BNO055 configured and its orientation. The axis orientation of the BNO055 can be re-configured to the new reference axis before experiment. (c) Illustration of roll, pitch, and yaw in terms of direction and rotation. Zoomed-in figures shows the example roll (-10.8°), pitch (21.5°), and yaw (-6°) extracted from the recorded quaternion value ($\boldsymbol{q_w}$, $\boldsymbol{q_x}$, $\boldsymbol{q_y}$, $\boldsymbol{q_z}$), when the rat is passing x=0, running from north to the south on the track. (d) Example path plot over time of a rat as they explore the rectangular track. Scatter color overlay corresponds to the yaw (head azimuth) of the BNO055 on the MiniLFOV. (e) Singular example as a rat makes a journey to all four corners of the environment and demonstrates a full range of head angles. (f) Polar distributions of sensor values for yaw (left), roll (middle), and pitch (right) during periods of time when the rats occupy the corners or make beelines through the direct path connecting the corners to retrieve reward, separated by which corner they end in (North vs. South journeys). (g) Average head angle (yaw) by position on the direct arm during northern and southern beelines for n=19 sessions from 3 rats. Mean +/- 1SD are plotted on the left for corner occupancy head angle. Only sessions with at least 8 beelines for a given direction are included. (h) Standard deviation of head angle by position on the direct path for the same n=19 sessions. Mean +/- 1SD for corner head angle standard deviations for these sessions are plotted in black.

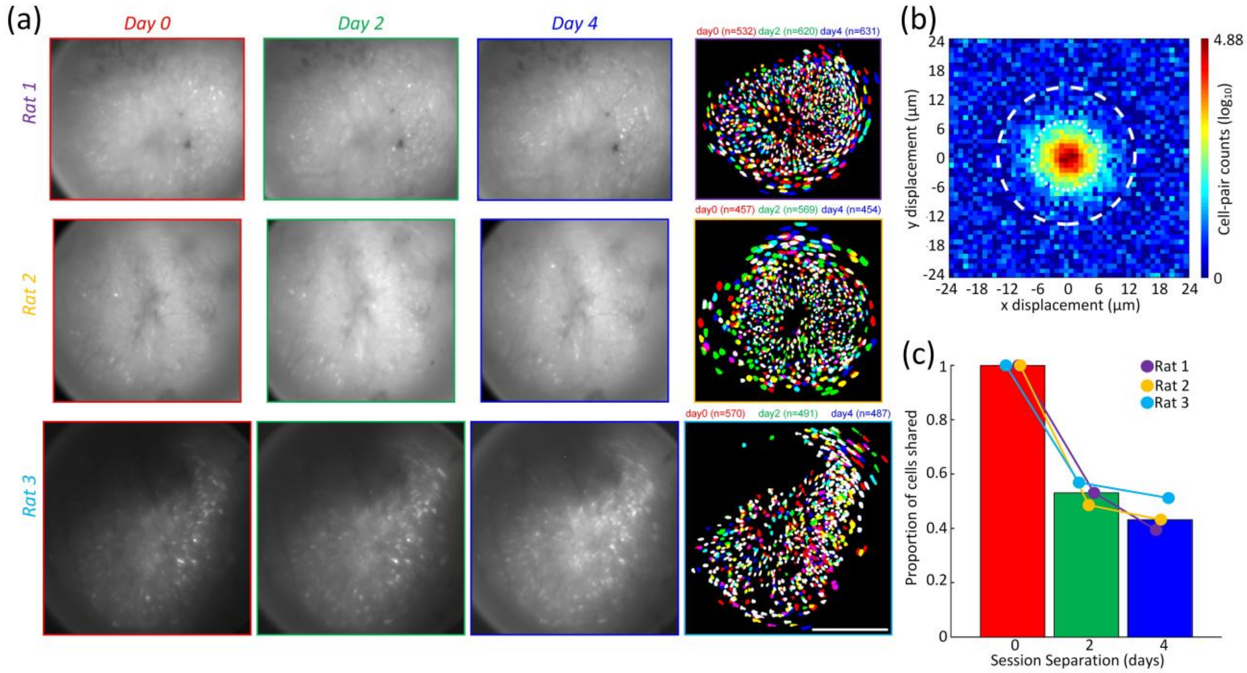

Fig. S11. Cells can be reliably recorded across sessions. Three imaging sessions were performed in the same task with 48-hours separating each recording to demonstrate that the MiniLFOV is capable of consistently recording activity from the same population of cells across multiple sessions. (a) Mean frames (Column 1-3) and extracted contours of cells (Column 4) across all three recording days from Rat 1 (N_0=532, N_2=620, N_4=631), Rat 2 (N_0=457, N_2=569, N_4=454), and Rat 3 (N_0=570, N_2=491, N_4=487). The contours of cells are colored by the recording day (day0=red, day2=green, day4=blue). The algorithm to match cells across all sessions is in Methods. Scale bar: 500 µm. (b) Distribution of centroid displacements for all cell pairs within a 24-µm radius, showing a large population of cells within the same location after across-day alignment. Distributions of centroid distances and spatial correlation for all cell pairs within 24 µm were computed, yielding a two-dimensional distribution that can be modeled and given a probability threshold to match cell pairs. Cell pairs with a probability >0.5 for both the centroid and correlation distributions were matched (low centroid distance and high spatial correlation), which is expected when recording the same cell. (c) Population overlap (the number of cells shared between sessions) the average population correlation vector between recording sessions decreased as a function of time from Rat 1 (day0=100%, day2=53.1%, day4=39.5%), Rat 2 (day0=100%, day2=48.6%, day4=43.2%), and Rat 3 (day0=100%, day2=56.9%, day4=51.2%).

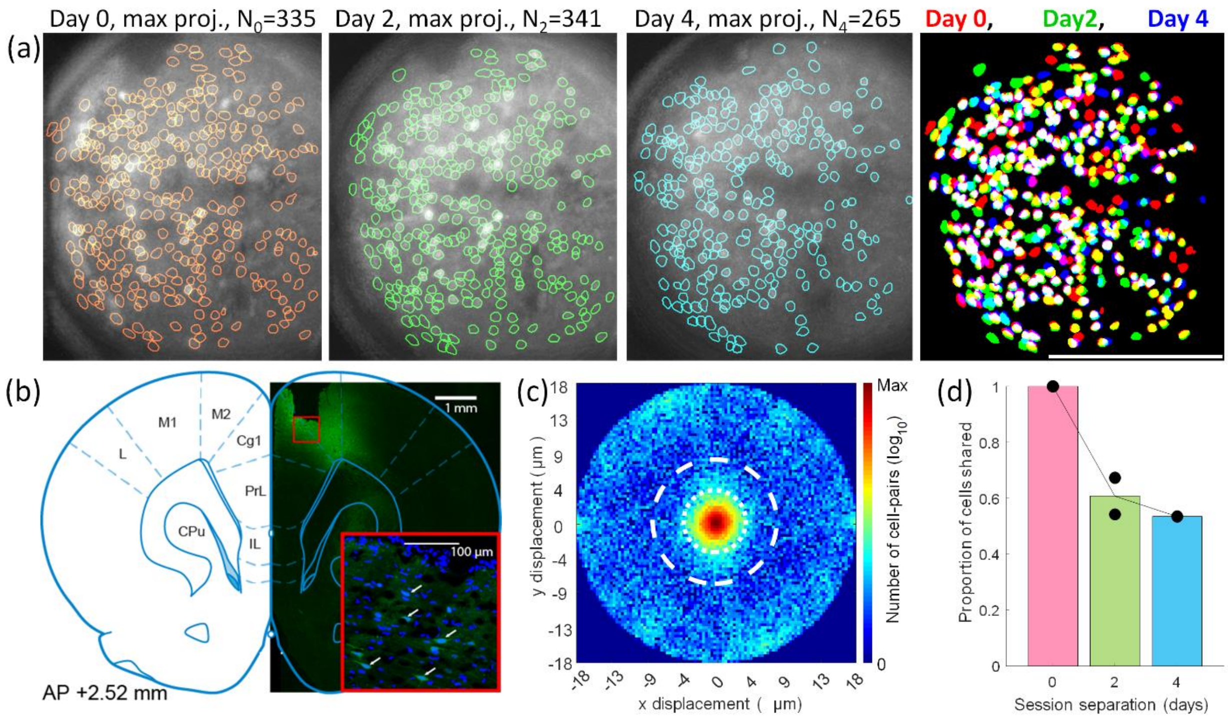

Fig. S12. Cells recorded from the anterior cingulate cortex (ACC) in a freely behaving rat can be reliably recorded across sessions. Three imaging sessions were performed in the same task with 48-hours separating each recording to demonstrate that the MiniLFOV is capable of consistently recording activity from the same population of cells across multiple sessions in the cortex. (a) Maximum frames with contours of extracted cells (Column 1-3) and contours of cells (Column 4), colored by the recording day (day0=red, day2=green, day4=blue), across all three recording days (N_0=335, N_2=341, N_4=265). The algorithm to match cells across all sessions is in Methods. Scale bar: 500 µm. (b) Example histology with a DAPI stain from Rat 1, showing GCaMP8m expression with the ACC layer beneath the implanted lens location. (c) Distribution of centroid displacements for all cell pairs within an 18-µm radius, showing a large population of cells within the same location after across-day alignment. Distributions of centroid distances and spatial correlation for all cell pairs within 18 µm were computed, yielding a two-dimensional distribution that can be modeled and given a probability threshold to match cell pairs. Cell pairs with a probability >0.5 for both the centroid and correlation distributions were matched (low centroid distance and high spatial correlation), which is expected when recording the same cell. (d) Population overlap (the intersection of cells identified and matched) between the 3 sessions. The average population overlap between recording sessions decreased as a function of time (2 days apart=0.60, 4 days apart=0.53).

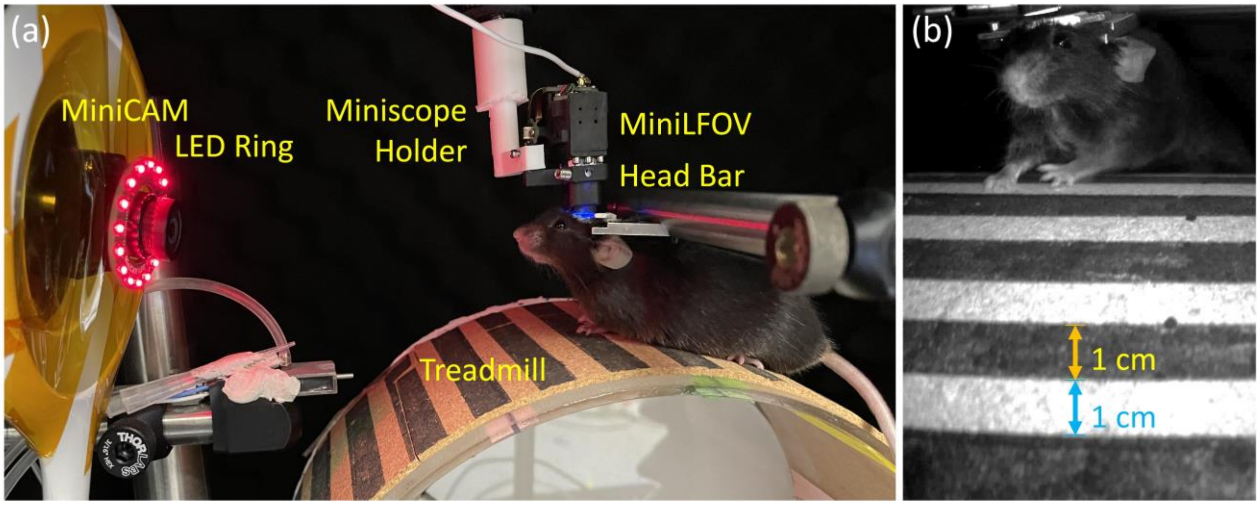

Fig. S13. Experimental setup for Ca^{2+} imaging of dorsal cortex through a cranial window in head-fixed mice. (a) Experimental setup. The mice were head-fixed atop a circular treadmill (22.9-cm diameter and the white and dark bars with 1-cm wide). The MiniLFOV was attached to an articulated ball-head holder mounted on a 3D translation stage built of three linear translation stages (XR25C/M, Thorlabs) so that the lateral position and height of the MiniLFOV relative to the cranial window can be adjusted. A MiniCAM is used to capture the movement speed of the mice and the red LED ring is used for illumination in dark environment. (b) Single frame taken by the MiniCAM.

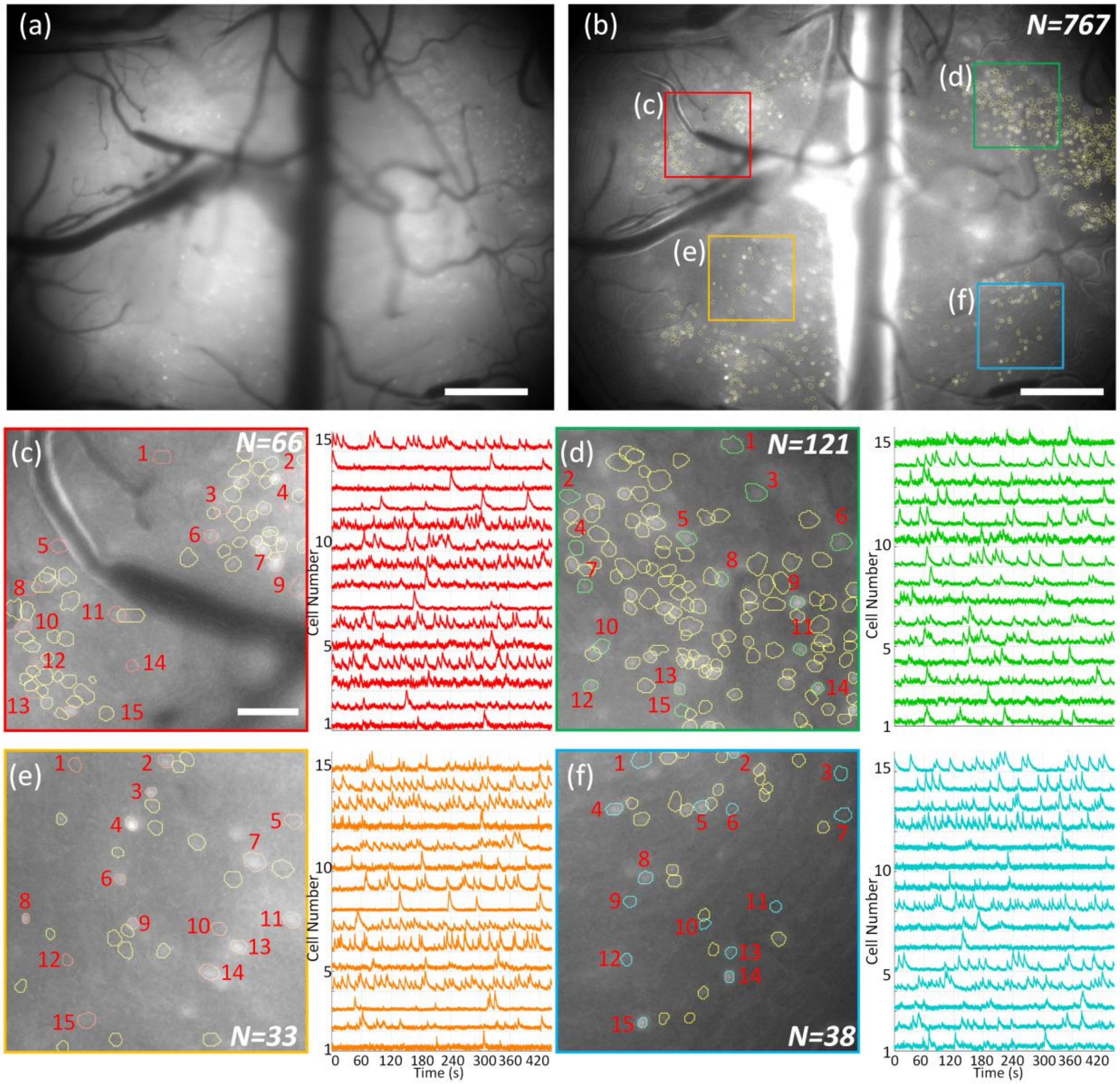

Fig. S14. Ca^{2+} imaging of dorsal cortex through a midcranial window in an additional head-fixed mouse. (a) Maximum projection from a 8-minute recording session after motion correction. Scale bar: 500 µm. (b) Maximum-intensity projection image of the raw video after motion correction and background removed with contours of 767 extracted cells from CNMF-E analysis via CaImAn circled in yellow. The colored boxes indicate four sub-regions which are zoomed in and shown in c-f. Scale bar: 500 µm. (c) Map of 66 cells in the red-boxed area in b and 15 randomly chosen cells with their low-pass filtered Ca^{2+} transients for the numbered cells are shown in red. Scale bar, 100 µm. (d) Map of 121 cells in the green-boxed area in b and 15 randomly chosen cells with their low-pass filtered Ca^{2+} transients for the numbered cells are shown in green. (e) Map of 33 cells in the orange-boxed area in b and 15 randomly chosen cells with their low-pass filtered Ca^{2+} transients for the numbered cells are shown in red. (f) Map of 38 cells in the cyan-boxed area in b and 15 randomly chosen cells with their low-pass filtered Ca^{2+} transients for the numbered cells are shown in cyan.

Table S1. Three configurations of MiniLFOV.

Configurations	Specifications									
	**L×W×H (mm)**	**Weight (g)**	**FOV (mm^2)**	**Resolution (μm)**	**WD (mm)**	**M**	**Pixel**	**Frame rate (FPS)**	**Clock (MHz)**	**Time**
Wire/WD: 1.8 mm 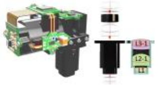	35×20×35	14	3.1×2.3	2.5-4.4	1.8	1.86	2592 × 1944	11	66.67	Long
		14	3.1×2.3	4.4-6.2	1.8	1.86	1296 × 972	23	66.67	Long
Wire/WD: 3.5 mm 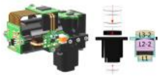	35×20×30	14	3.6×2.7	3.5-6.2	3.5	1.56	2592 × 1944	11	66.67	Long
		14	3.6×2.7	6.2-7.8	3.5	1.56	1296 × 972	23	66.67	Long
Wirefree 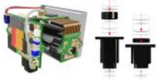	35×20×35<br>35×20×30	25	1.4×1.4<br>1.7×1.7	4.4-6.2<br>6.2-7.8	1.8<br>3.5	1.86<br>1.56	608 × 608	20	24	≤ 1 hour

Table S2. List of large FOV microscopes.

Miniscopes	Weight	FOV	Resolution	WD	M	Single Cell Resolution
Mini-mScope(*33*)	3.8 g	8 × 10 mm^2	39 to 56 μm	×	0.36-0.45×	No
cScope(*34*)	35 g	7.8 × 4 mm^2	14 μm	×	0.7×	No
Boston Miniscope(*26*)	4.2-4.5 g	2.7 × 1.8 mm^2	7 μm	~2.5 mm	~1.3×	Yes
Kiloscope(*27*)	1.4 g	4.8 × 3.6 mm^2	~10 μm	1 mm	~1×	Yes
MiniLFOV	13.9 g	3.1 × 2.3 mm^2<br>3.6 × 2.7 mm^2	2.5 μm<br>3.5 μm	1.8 mm<br>3.5 mm	1.86×<br>1.56×	Yes

The goal of our proposed MiniLFOV is to develop a more stable, sensitive, and easy to access platform which can be broadly used in neuroscience research. Instead of prototype, proof-of-concept or custom design, MiniLFOV is designed with off-the-shelf lenses which are easy to get from Edmund optics. The weight is compromised to get a higher NA both for excitation and fluorescence collection to make it more sensitive than other large FOV counterparts. Higher sampling rate can be obtained with higher optical magnification.

Table S3. List of components for MiniLFOV assembly.

	Component	Quantity	Vendor	Part #	Weblink
	Optics	11			
L1	6.25mm Dia. x 60mm FL, MgF2 Coated, Achromatic Doublet Lens	1	Edmund Optics	#45-345	https://www.edmundoptics.com/p/625mm-dia-x-60mm-fl-mgfsub2sub-coated-achromatic-doublet-lens/5815/
L2-1	9mm Diameter x 12mm EFL Aspherized Achromatic Lens	1	Edmund Optics	#49-656	https://www.edmundoptics.com/p/9mm-diameter-x-12mm-efl-aspherized-achromatic-lens/10162/
L3-1	9mm Dia. x 27mm FL, MgF2 Coated, Achromatic Doublet Lens	1	Edmund Optics	#45-092	https://www.edmundoptics.com/p/9mm-dia-x-27mm-fl-mgfsub2sub-coated-achromatic-doublet-lens/5583/
L2-2	9mm Diameter x 18mm EFL Aspherized Achromatic Lens	1	Edmund Optics	#49-657	https://www.edmundoptics.com/p/9mm-diameter-x-18mm-efl-aspherized-achromatic-lens/10163/
L3-2	9mm Dia. x 36mm FL, MgF2 Coated, Achromatic Doublet Lens	1	Edmund Optics	#45264	https://www.edmundoptics.com/p/9mm-dia-x-36mm-fl-mgfsub2sub-coated-achromatic-doublet-lens/5748/
L4	12.5mm Diameter x 14mm EFL Aspherized Achromatic Lens	1	Edmund Optics	#49-658	https://www.edmundoptics.com/p/125mm-diameter-x-14mm-efl-aspherized-achromatic-lens/10164/
L5	12.0mm Diameter x -48 FL, MgF2 Coated, Plano-Concave Lens	1	Edmund Optics	#45-019	https://www.edmundoptics.com/p/120mm-diameter-x48-fl-mgfsub2sub-coated-plano-concave-lens/5531/
ExF	$4\times4\times1$ mm^3	1	Chroma	ET470/40x	https://www.chroma.com/products/parts/et470-40x
DM	$14\times10\times1$ mm^3	1	Chroma	T500spxr	*Ask for quote*
EmF	$10\times10\times1$ mm^3	1	Chroma	ET525/50m	https://www.chroma.com/products/parts/et525-50m
EWL	A-58N with an Anti-Reflective coating optimized in the visible range	1	Varioptics/Corning	Corning Arctic 58N	https://www.corning.com/worldwide/en/products/advanced-optics/product-materials/corning-varioptic-lenses/corning_varioptic_lenses_introduces_new_a-

					58n_variable_focus_liquid-lens.html
CMOS Sensor		1			
CMOS	1/2.5-Inch 5 Mp CMOS Digital Image Sensor, Onsemi	1	Digikey	488-MT9P031I12STM-DP-ND	https://www.digikey.com/en/products/detail/onsemi/MT9P031I12STM-DP/10447064 https://www.onsemi.com/pdf/datasheet/mt9p031-d.pdf
Excitation LEDs		2			
	LED LUXEON Z BLUE 470NM 2SMD	2	Digikey	LXZ1-PB01	https://www.digikey.at/product-detail/en/lumileds/LXZ1-PB01/1416-1052-1-ND/3961157
	Head orientation Senor (BNO055)				
	Accelerometer, Gyroscope, Magnetometer, 9 Axis Sensor I²C, UART Output	1	Digikey	828-1058-1-ND	https://www.digikey.com/en/products/detail/bosch-sensortec/BNO055/6136301
3D Printer					
High-end Stereolithographic (SLA) printer		1	Formlabs	Form 3	https://formlabs.com/3d-printers/form-3/
Housing					
Objective module body		1	N/A	N/A	https://github.com/Aharoni-Lab/Miniscope-LFOV/blob/main/3D-Printed-Bodies_MiniLFOV/Objective-Module_Body_MiniLFOV.stl
Spacer		1	N/A	N/A	https://github.com/Aharoni-Lab/Miniscope-LFOV/blob/main/3D-Printed-Bodies_MiniLFOV/Objective-Spacer_MiniLFOV.stl
Emission module body		1	N/A	N/A	https://github.com/Aharoni-Lab/Miniscope-LFOV/blob/main/3D-Printed-Bodies_MiniLFOV/Emissi

				on-Module-Body_MiniLFOV.stl
Cover	1	N/A	N/A	https://github.com/Aharoni-Lab/Miniscope-LFOV/blob/main/3D-Printed-Bodies_MiniLFOV/Cover_MiniLFOV.stl
Cable Holder	1	N/A	N/A	https://github.com/Aharoni-Lab/Miniscope-LFOV/blob/main/3D-Printed-Bodies_MiniLFOV/Cable-Holder_MiniLFOV.stl
L5 Lens Holder	1	N/A	N/A	https://github.com/Aharoni-Lab/Miniscope-LFOV/blob/main/3D-Printed-Bodies_MiniLFOV/L5-Holder_MiniLFOV.stl
CMOS module body	1	N/A	N/A	https://github.com/Aharoni-Lab/Miniscope-LFOV/blob/main/3D-Printed-Bodies_MiniLFOV/CMOS-Module-Body_MiniLFOV.stl
Circuits				
MiniLFOV Circuit	1	N/A	N/A	https://github.com/Aharoni-Lab/Miniscope-LFOV
Screws				
M1 thread-forming screws	12	McMaster-Carr	96817a704	https://www.mcmaster.com/tapping-screws/screw-size~m1/
18-8 Stainless Steel Socket Head Screw, 0-80 Thread Size	8	McMaster-Carr	92196A052	https://www.mcmaster.com/catalog/92196a052
4-40 Stainless Steel Setscrew, 1/4" Long	2	Thorlabs	SS4S025	https://www.thorlabs.com/thorproduct.cfm?partnumber=SS4S025
Cabling & Hardware Control	1			
50 Ω, flexible coaxial cable	1	Cooner Wire	CW2040-3650SR	https://www.coonerwire.com/mini-coax/

Data acquisition (DAQ) device	1	Open Ephys	DAQ v3.3	https://open-ephys.org/miniscope-daqs/daq http://miniscope.org/index.php/Main_Page
Wire-free DAQ	1	N/A	N/A	https://github.com/Aharoni-Lab/Miniscope-LFOV
SparkFun Infrared Control Kit	1	Mouser	474-KIT-14677 KIT-14677	https://www.mouser.com/ProductDetail/sparkfun-electronics/kit-14677/?qs=l7cgNqFNU1jwLmEywj2NAQ%3D%3D&countrycode=US&currencycode=USD https://www.sparkfun.com/products/14677
EMU FOR SAM AND AVR MCU KIT	1	Digikey	ATATMEL-ICE-ND	https://www.digikey.com/en/products/detail/microchip-technology/ATATMEL-ICE/4753379
Firmware & Software				
DAQ firmware for UCLA Miniscope platforms	N/A	N/A	N/A	https://github.com/Aharoni-Lab/Miniscope-DAQ-Cypress-firmware
Aharoni-Lab Miniscope-DAQ-QT-Software	N/A	N/A	v1.11	https://github.com/Aharoni-Lab/Miniscope-DAQ-QT-Software
Wire-free DAQ firmware	N/A	N/A	N/A	https://github.com/Aharoni-Lab/Miniscope-Wire-Free-DAQ/tree/master/MCU-firmware
microSD configuration	N/A	N/A	N/A	https://mh-nexus.de/en/hxd/
MiniCAM				
Open-source behavioral camera for imaging freely behaving animals in behavioral and neuroscience experiments	1	Open Ephys	N/A	https://github.com/Aharoni-Lab/MiniCAM https://open-ephys.org/minicam/minicam
Piplines				
CaImAn	N/A	N/A	N/A	https://github.com/flatironinstitute/CaImAn
OASIS	N/A	N/A	N/A	https://github.com/zhoupc/OASIS_matlab
Wire-free Data read from microSD	N/A	N/A	N/A	https://github.com/Aharoni-Lab/Miniscope-Wire-Free-

	DAQ/tree/master/Wire-Free-DAQ-Software

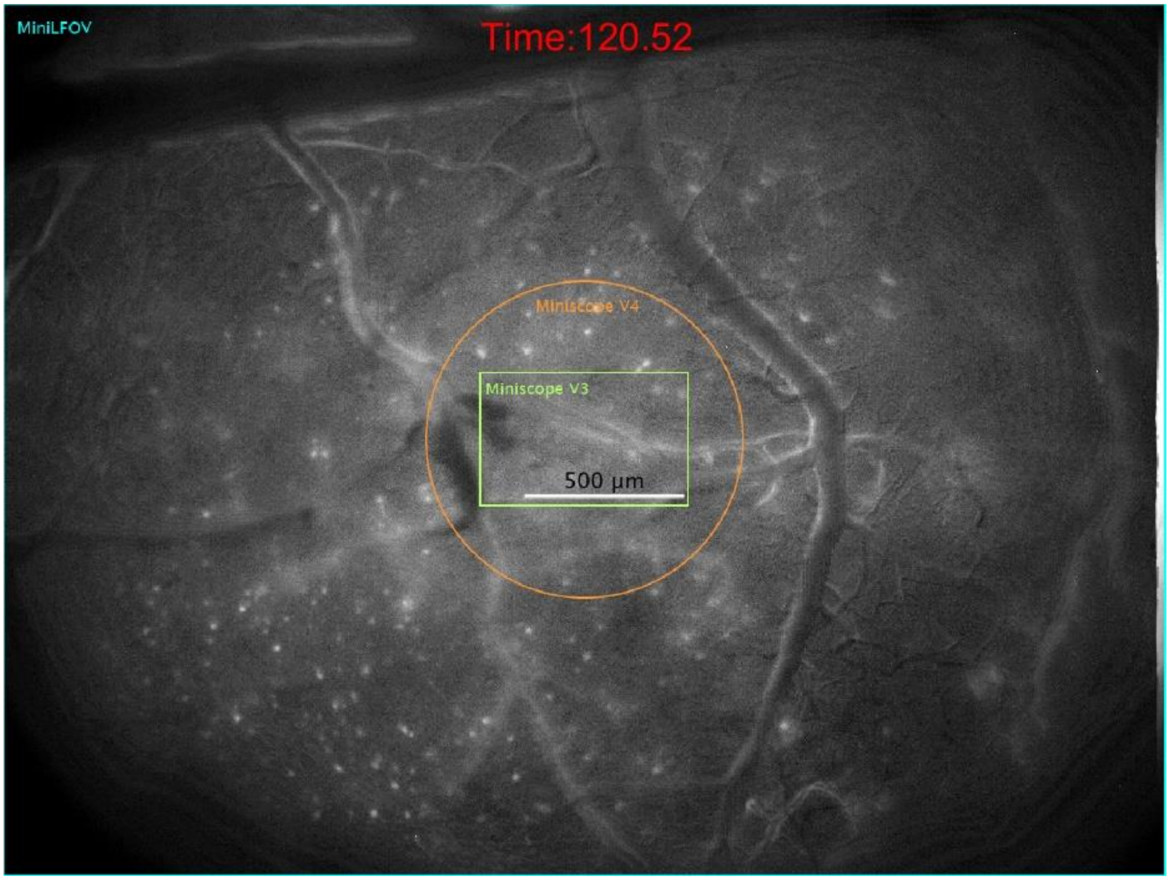

Movie S1. Field of view (FOV) size comparison of Miniscope V3, Miniscope V4, and MiniLFOV. MiniLFOV has 30-fold increase in FOV than the previous generation UCLA Miniscope V3 and 12-fold increase in FOV than the current generation UCLA Miniscope V4. The background was calculated as the minimum projection of the raw video (after motion correction) and removed to show clear neuronal activities in this video.

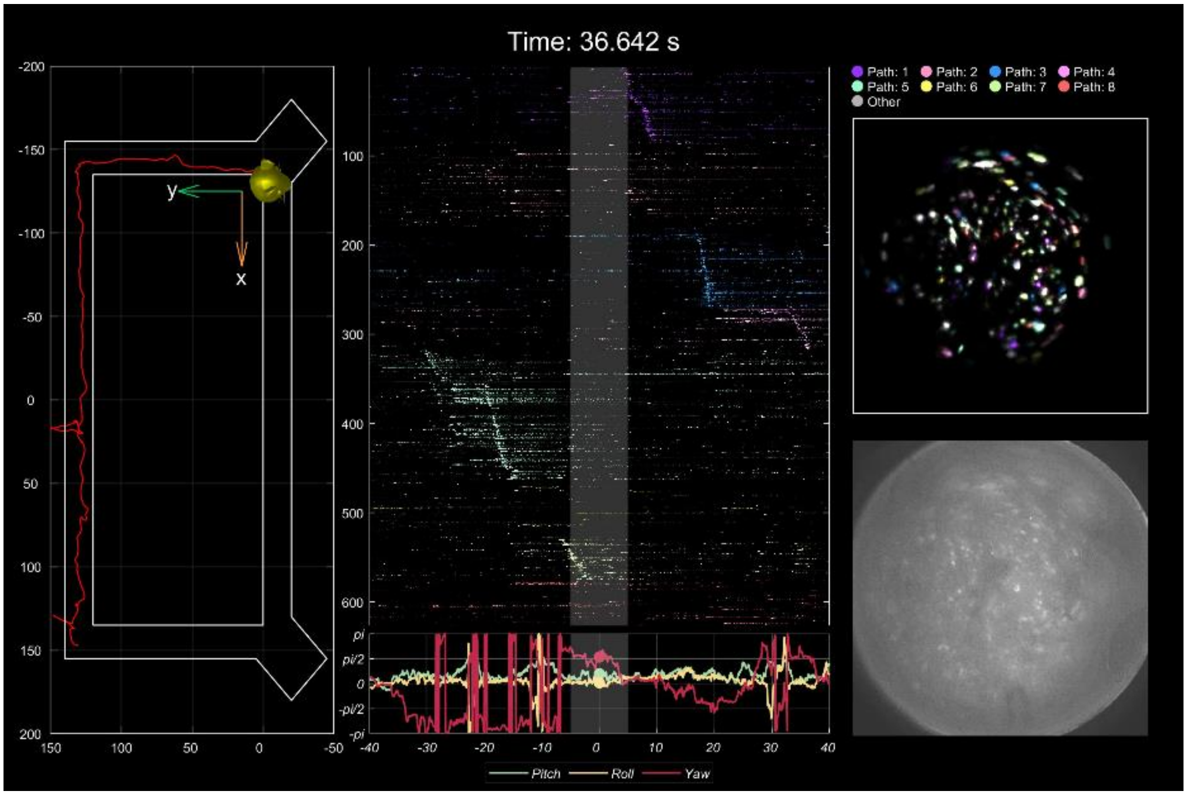

Movie S2. An example video showing the position of the rat, head orientation, and place cell activity during behavior. To record place cell activity from the dorsal CA1 region in rats, we imaged Ca^{2+} dynamics in GCaMP7s-expressing neurons in dorsal CA1 while rats performed navigation of a rectangular track environment. We present one exemplary session of one rat to demonstrate the effectiveness of the system. Recordings took place on a rectangular track (2.5 m × 1.25 m), with two reward feeders on two corners of the track. Positions on the track and head orientation of the rat were extracted from a software synchronized behavioral camera and on-board head orientation sensor respectively. The raw video is 972 × 1296 pixels after within-sensor 2x pixel binning and then further cropped to the pixel region containing the relay GRIN lens (720 × 720 pixels). In this example recording, a total number of 1357 cells can be detected and extracted using CNMF-E analysis via CaImAn. As expected, a significant population of the cells (626; 46.2%) are spatially tuned along at least one direction on the 4 arms on the rectangular track. The behavior of the rat was serialized into 8 paths (4 arms with 2 running directions). Place cells recorded during the session were significantly modulated by both position and direction of travel and span across all regions of the track (**middle**). The raw video after motion correction shows as a reference (**bottom right**) for the video showing the activity of the extracted cells (**top right**, marked in 9 colors to represent the places cell for each path and other cells with spatial information below 95% of 500 randomly shifted deconvolved neural activity for each cell). These data demonstrate how the MiniLFOV can improve experimental efficacy by yielding more cells in larger research models, broadening the horizon of potential hypotheses for researchers.

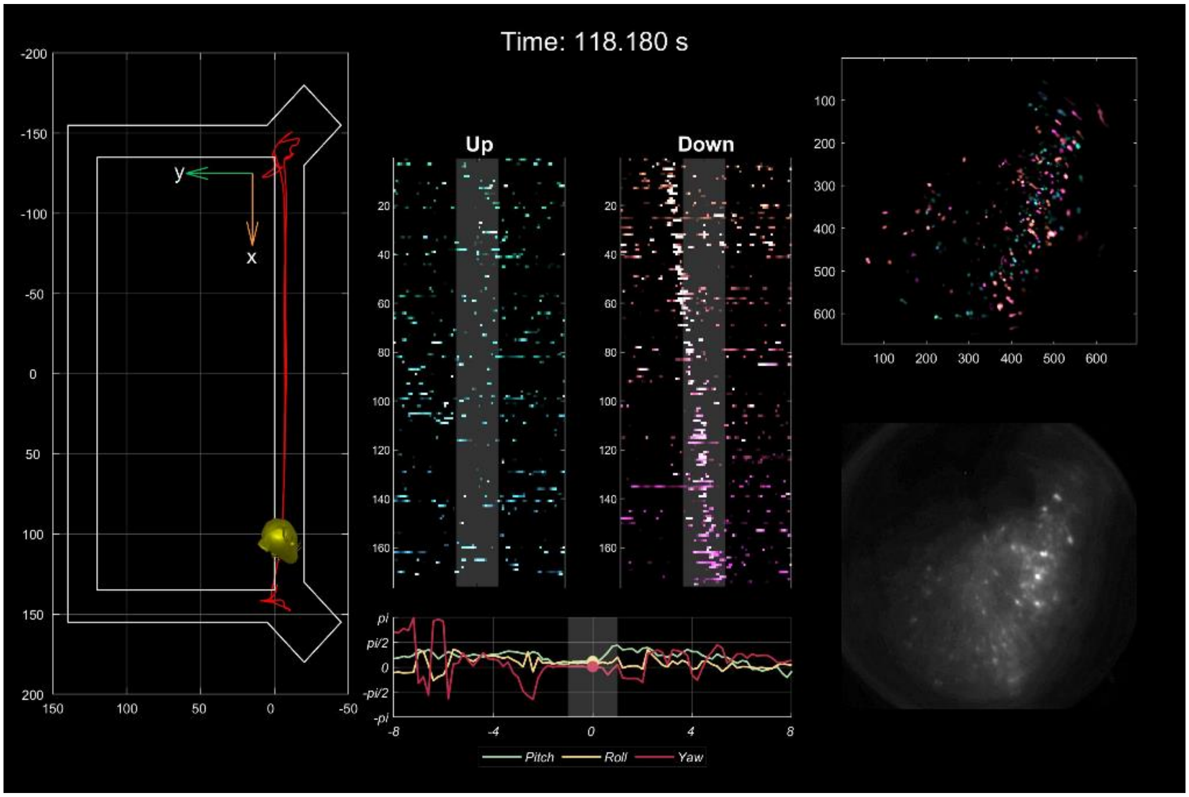

Movie S3. An example video showing the position of the rat, head orientation, and place cell activity during behavior. In this video, only two directions (Up and Down) on the long arm with two reward feeders on two corners are considered and plotted. Place cells related to running up and down are shown in the middle of the video.

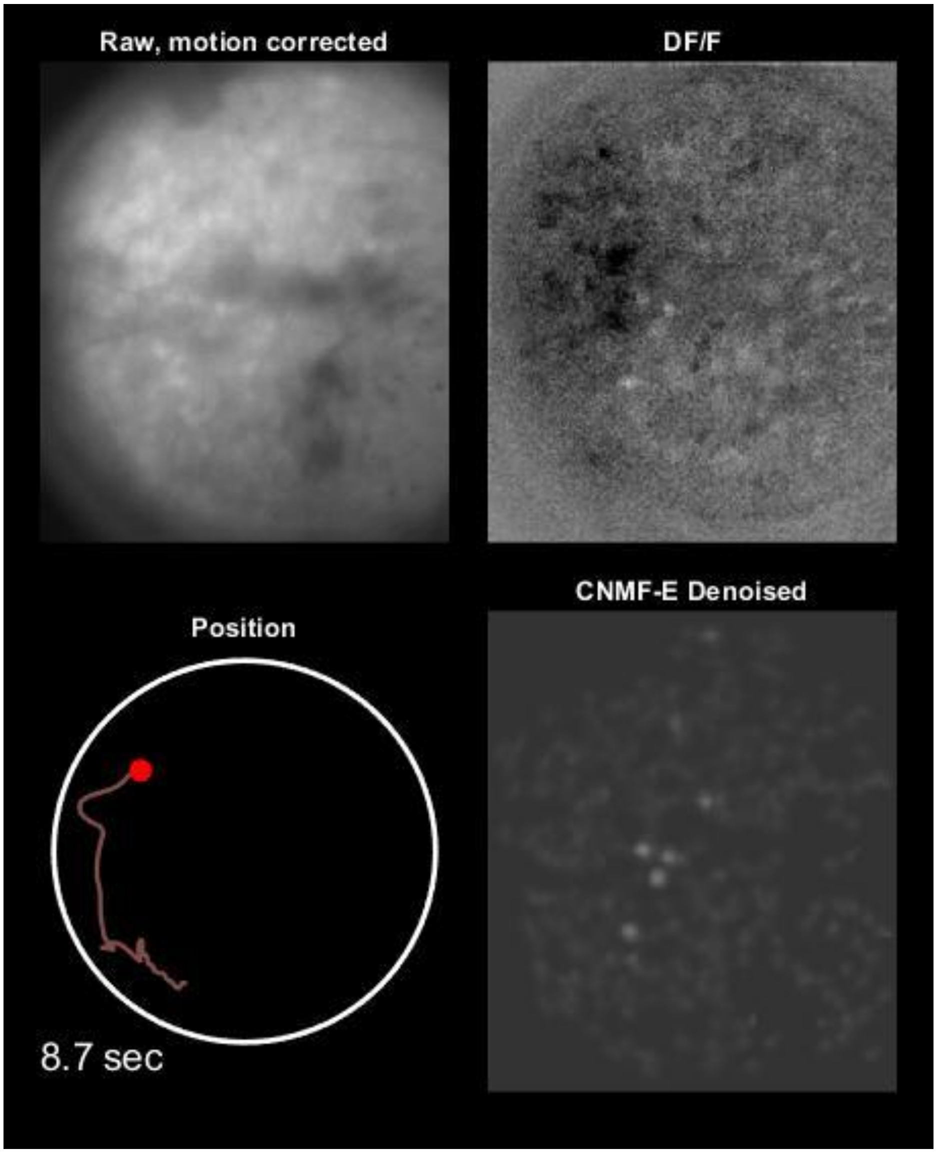

Movie S4. An example video showing imaging of GCaMP8m expressing neurons through a 1mm GRIN implanted in rat ACC. Raw motion corrected video (top left), DF/F (top right), and extracted denoised fluorescence from CaImAn ($C_{(t)}$*A, bottom right) are shown alongside the position of the rat (bottom left) as they explore an open field environment.
